# Platinum-based drugs induce phenotypic alterations in nucleoli and Cajal bodies in prostate cancer cells

**DOI:** 10.1186/s12935-023-03205-0

**Published:** 2024-01-13

**Authors:** Enkhzaya Batnasan, Minttu Kärkkäinen, Sonja Koivukoski, Nithin Sadeesh, Sylvain Tollis, Pekka Ruusuvuori, Mauro Scaravilli, Leena Latonen

**Affiliations:** 1 Institute of Biomedicine, University of Eastern Finland, 1627, 70211 Kuopio, Finland; 2 Institute of Biomedicine, University of Turku, Turku, Finland

**Keywords:** Nucleoli, Cajal bodies, Prostate cancer, Platinum drugs, Stress response, Nucleus

## Abstract

**Purpose:**

Platinum-based drugs are cytotoxic drugs commonly used in cancer treatment. They cause DNA damage, effects of which on chromatin and cellular responses are relatively well described. Yet, the nuclear stress responses related to RNA processing are incompletely known and may be relevant for the heterogeneity with which cancer cells respond to these drugs. Here, we determine the type and extent of nuclear stress responses of prostate cancer cells to clinically relevant platinum drugs.

**Methods:**

We study nucleolar and Cajal body (CB) responses to cisplatin, carboplatin, and oxaliplatin with immunofluorescence-based methods in prostate cancer cells. We utilize organelle-specific markers NPM, Fibrillarin, Coilin, and SMN1, and study CB-regulatory proteins FUS and TDP-43 using siRNA-mediated downregulation.

**Results:**

Different types of prostate cancer cells have different sensitivities to platinum drugs. With equally cytotoxic doses, cisplatin, and oxaliplatin induce prominent nucleolar and CB stress responses while the nuclear stress phenotypes to carboplatin are milder. We find that Coilin is a stress-specific marker for platinum drug response heterogeneity. We also find that CB-associated, stress-responsive RNA binding proteins FUS and TDP-43 control Coilin and CB biology in prostate cancer cells and, further, that TDP-43 is associated with stress-responsive CBs in prostate cancer cells.

**Conclusion:**

Our findings provide insight into the heterologous responses of prostate cancer cells to different platinum drug treatments and indicate Coilin and TDP-43 as stress mediators in the varied outcomes. These results help understand cancer drug responses at a cellular level and have implications in tackling heterogeneity in cancer treatment outcomes.

**Supplementary Information:**

The online version contains supplementary material available at 10.1186/s12935-023-03205-0.

## Introduction

Chemotherapy with anti-cancer drugs aims to stop the growth of cancer cells either by killing them or by stopping them from dividing. Chemotherapeutic drugs are often targeted to damage DNA to prevent use of it in transcription and DNA replication, leading to replication crisis and/or programmed cell death, apoptosis. Platinum compounds are chemotherapeutic drugs that form covalent adducts with cellular DNA resulting in DNA damage through which they exert their anti-tumour activity. The chemotherapeutic agent cisplatin has been used in the clinics for already 50 years. While it has been effective in treatment against many cancers, the patient responses vary and adverse effects are significant, which has seeded numerous attempts to find better platinum-based analogues. Of these, two other compounds—carboplatin and oxaliplatin—have met FDA standards for medical use. While cisplatin and carboplatin are used with e.g. breast and lung malignancies, oxaliplatin is mainly used in colorectal and other gastrointestinal cancers. While incompletely understood currently, the different specificities in inducing cellular and damage responses may underlie the preferential effectiveness against different types of cancers. There is evidence that at clinically relevant doses, cisplatin kills cells via the canonical DNA damage response (DDR), while oxaliplatin induces disruptions also in ribosome biogenesis [3, 21]. Proteomic profiling has shown that oxaliplatin downregulates centrosomal proteins, RNA processing, and ribosomal proteins, supporting the idea that after the initial DNA damage response, nucleolar and ribosomal stress are triggered [14]. Also other platinum compounds can induce nucleolar stress, although the extent can vary [22].

Nucleolar stress reflects inhibition of transcription by RNA polymerase I and downregulation of ribosome synthesis, evidenced by a decrease in transcription of ribosomal genes, dispersal of the nucleolar components to nucleoplasm, translocation of many factors to nucleoli, and formation of nucleolar caps in the outer surface of the nucleoli facing nucleoplasm [1, 13, 25]. Nucleolar stress can be monitored through translocation of NPM to nucleoplasm, reflecting the dispersal of the phase-separated nucleolar structures following inhibition [3, 4, 10, 12, 17, 22]. The nucleolus is, however, not the only phase-separated nuclear organelle that may respond to DNA damage and inhibition of transcription. Cajal bodies (CBs) are key nuclear bodies in RNA processing, yet their responses to cytotoxic drugs remain mostly unstudied. The CBs are involved in, e.g., RNA biogenesis, the assembly, maturation, and recycling of small nuclear ribonucleoprotein (snRNPs), histone mRNA processing, and telomere maintenance [15]. Recent evidence suggests that CBs are stress-responsive and fluctuate in number and size upon specific stimuli or genomic activity [7]. Coilin, a key component of CBs that has a role in snRNP biogenesis, has been shown to participate in downregulation of RNA polymerase I activity upon cisplatin-induced DNA damage [6], but to the best of our knowledge, Coilin responses to carboplatin and oxaliplatin have not been described.

Prostate cancer is one of the leading cancer types in men in Western countries [16], with surgery or radiation therapy being effective against organ-confined cancer. For advanced forms of the disease, androgen deprivation therapy (ADT), targeting the activity of androgen receptor (AR) that is driving the cancer, is initially effective. Eventually, however, ADT fails and castration resistant prostate cancer (CRPC) emerges [24]. Platinum-based compounds have been studied in advanced prostate cancer patients in many clinical trials and, overall, they have demonstrated moderate to good anti-tumour activity in men with advanced CRPC [18]. They can be used as monotherapy or in combination with other chemotherapy agents mainly for CRPC but also with primary PC, although the optimal compounds, dosing regimens, and potential combination partners may yet to be identified [8]. There is evidence to show that the heterogeneity in prostate cancer affects the success of the use of platinum compounds [8], but the determinants of this at the cellular level remain to be identified. Hence, increased understanding of molecular and cellular events behind the heterogeneity is needed to select prostate cancer patients most likely to benefit from platinum-based therapy [18].

Here, we set out to assess heterogeneity in nuclear stress responses to platinum compounds in prostate cancer cells. We study the responses to the platinum-based chemotherapeutic agents in clinical use, namely cisplatin, carboplatin and oxaliplatin, using three different prostate cancer cells lines representing different biological types of advanced prostate cancer (AR positive, AR negative, and expressing AR transcript variant). We screen for induced effects on nucleoli and CBs, quantify the cellular phenotypes, and indicate molecular determinants for the responses. We find heterologous responses to the platinum-based drugs that are associated with prostate cancer cell types with different AR status, and we identify Coilin and TDP-43 as prominent platinum-responsive nuclear proteins. Our findings indicate variability of prostate cancer cells’ response to chemotherapy and have implications in future therapy regimen considerations.

## Materials and methods

### Cell culture

LNCaP, 22Rv1, and PC-3 cells were purchased from the American Type Culture Collection (ATCC, Manassas VA, USA). Cells were grown RPMI 1640 medium (Lonza, Verviers, Belgium) containing with 10% fetal bovine serum (Gibco), 1% penicillin streptomycin (Gibco) and 1% L -glutamine (Gibco). All cells were maintained in 5% CO_2_ and 100% humidity at 37 °C.

### Antibodies and reagents

Cells were treated with Oxaliplatin (NSC 266046, Selleck Chemistry, Houston, TX, USA), Cisplatin (Calbiochem, Sigma Aldrich), Carboplatin (Tocris) and Actinomycin D (Sigma Aldrich) in different dosages for 24 h. The primary antibodies used were anti-NPM mouse monoclonal antibody (E-3, Santa Cruz Biotechnology, CA, USA), anti-Coilin rabbit monoclonal antibody (D2L3J, Cell Signaling Technology, Leiden, The Netherlands), anti-SMN1 mouse monoclonal antibody (2F1, Cell Signaling Technology), anti-Fibrillarin rabbit monoclonal antibody (C13C3, Cell Signaling Technology), anti-FUS mouse monoclonal antibody (4H11, Santa Cruz Biotechnology), anti-TARDBP (TDP43) mouse monoclonal antibody (2E2-D3, Abnova), anti-actin mouse monoclonal (ACTN05 C4, Abcam, Cambridge, UK), anti-tubulin mouse monoclonal antibody (TU-02, Santa Cruz Biotechnology) and anti-lamin rabbit polyclonal antibody (B1, ab16048, Abcam).

### Cell density assay

PC-3, LNCaP, and 22Rv1 cells were seeded at 5000, 10,000, and 7500 cells per well, respectively. The following day, the indicated drugs were added and the cells were then monitored by phase-contrast imaging using IncuCyte^®^ S3 Live-Cell Analysis system (Sartorius AG, Göttingen, Germany). Cell confluency was analyzed using automated analysis in IncyCyte 2021C software (Sartorius AG).

### SiRNA Transfection

For western blotting and immunofluorescence experiments, cells were plated on a 10 cm dish with coverslips with 1 million cells per dish for PC-3 and 1.2 million cells for LNCaP. The cells were then reverse transfected with a 20 nM pool of 2 siRNAs with INTERFERin® transfection reagent (Polyplus Transfection SA, Illkirch, France) according to manufacturer’s recommendations. siRNAs targeting FUS and TDP-43 were obtained from ThermoFisher Scientific (Silencer^™^ Select Pre-Designed siRNA, cat# 4392420, FUS_1 ID: s5401; FUS_2 ID: s5403; TDP-43_1 ID: s23830; TDP-43_2 ID: s23829, Ambion, ThermoFisher Scientific, Waltham, Massachusetts, USA). Silencer^™^ Negative Control #1 (AM4635, Ambion, ThermoFisher Scientific) was used as control. After 72 h, the coverslips were fixed with 4% PFA for 30min in RT or cells were lysed for protein extraction. For cell density measurements, PC-3 and LNCaP cells were plated on a 96-well plate at confluence of 9000 cells and 16,000 cells per well, respectively. The cells were reverse transfected as above and the following day the medium was replaced with medium containing the indicated drugs. The cells were then monitored using IncuCyte® S3 Live-Cell Analysis system (Sartorius AG).

### Immunofluorescence staining

Prior to this study, seeding numbers per well had been tested and optimized, approximately ≈ 70% cell confluence for 22Rv1 and LNCaP cells meanwhile 80% cell confluence for PC-3 cells upon fixation. LNCaP cells were seeded at 40,000 cells, 22Rv1 cells at 35,000 cells, and PC-3 cells at 45,000 cells per well on glass coverslips (CB00130RAc20MNZO, Epredia Coverslips, Gerhard Menzel GmbH, Saarbruckener Str.248 38,116, Braunschweig, Germany) in 24 well plates. Cells were allowed to adhere overnight or LNCaP cells for 2 days before drug treatments for 24 h, after which immunofluorescence staining was performed. Cells were fixed with 4% paraformaldehyde (PFA, Sigma‐Aldrich, Darmstadt, Germany) for 30 min, permeabilized with phosphate‐buffered saline (PBS; 137 mM NaCl, 2.7 mM KCl, 10 mM Na2HPO4, 1.8 mM KH2PO4, pH 7.2) containing 0.5% NP-40 (Sigma‐Aldrich) for 30 min, blocked in 3% BSA in PBS for 30 min followed, incubated with primary antibody (used in concentrations of 2–4 μg/ml in blocking buffer) incubated in 4 °C overnight. For the immunostaining, the primary antibodies were used in blocking buffer (PBS + 3% BSA) followed by incubation with 1 μg/ml secondary antibodies (anti-rabbit Alexa Fluor 594 and anti-mouse Alexa Fluor 488; Cell Signaling Technology, Leidin, The Netherlands) in combination or single incubation for an hour at room temperature. Cell nuclei were counterstained with 300 nM 4ʹ,6‐diamidino‐2‐phenylindole (DAPI) followed by mounting of coverslips with Vectashield Mounting Medium (Vector Laboratories, Burlingame, CA, USA).

### Confocal microscopy and image acquisition

Image acquisition was performed using a Zeiss LSM 800 Airyscan confocal microscope equipped with a Plan-Apochromat 63 × /1.40 Oil DIC M27 immersion objective plus utilizing Zen Blue Application software using the following settings: Pinhole 1 Airy unit, 16-bit depth, scan speed 600 Hz, line average 8, and image size 2040 × 2040 (pixels). The gain (maximum 700 V) and exposure time was kept the same for all images for each experiment.

### Western blotting

Cells were lysed in Triton-X lysis buffer containing 50 mM Tris–HCl, pH 7.5, 150 mM NaCl, 0.5% Triton-X-100, 1 mM Phenylmethylsulfonyl fluoride, 1 mM DTT, and 1 × Complete protease inhibitor cocktail (Roche), after which cellular debris was removed by centrifugation. Isolated protein concentration was measured by Tecan Infinite M200 and used as equal concentrations in each gel. Samples were resuspended in 4 × Laemmli sample buffer and denatured at 100 °C. Proteins were separated by 10% sodium dodecyl sulfate–polyacrylamide gel electrophoresis (SDS-PAGE) gel and immobilized onto Nitrocellulose-membranes (Thermo Fisher Scientific). Primary antibody signals were detected with goat-anti-mouse or goat-anti-rabbit HRP-conjugated antibodies (Invitrogen, Thermo Fisher Scientific) and chemiluminescence reactions using Clarity Western ECL Substrate reagent (Bio-Rad Laboratories) detected using ChemiDoc MP Imaging system (Bio-Rad Laboratories). Representative blots of three replicate experiments are shown. Signal intensities were quantified with ImageJ for relative amounts as a ratio to loading control, and statistical significances of treated samples compared to control samples were assessed with two-tailed t-test.

### Slide scanner light microscopy

For quantifying the drug-induced nucleolar and CB morphology and distributions, immunofluorescence slides were scanned by Leica Thunder Imager 3D Tissue slide scanner (Leica Microsystems, Wetzlar, Germany) with HC PL FLUOTAR 63x/1.30 oil objective with 18 bit, 18 tiles, and 5 step Z stack unified in maximum projection, using Leica K5 sCMOS camera. Images were processed with Leica Application Suite X Software within Leica Slide Scanner supply package. All images were rescaled to 8 bit (dynamic range of 0–255) to reduce the file size. For each sample, at least 300 cells were analysed for manual phenotypic classification.

### Quantitation of Cajal bodies

Image analysis was performed using custom scripts in Matlab, including tools from the Image Processing Toolbox (Mathworks). The DAPI signal was used to detect cells’ nuclei and Coilin signal to detect CBs. In each channel, Matlab’s edge detection function implementing the Canny method was used to identify the contours of the regions of interest (ROIs) and morphological operations (isolated pixels cleaning, hole filling, morphological closing/opening) were performed to obtain the binary masks of the ROIs. Objects touching the border of the image, as well as very large nuclear detections (> 4000 pixels) and small nucleolar detections (< 50 pixels) were removed from further analysis, the former as they likely represent multiple nuclei detected as one, and the latter as they are imaging artefacts. No size-based selection was performed on Cajal bodies. Masks were visually compared with raw fluorescence data, and the edge-detection thresholds were adjusted to improve the quality of masking. The same thresholds were then used for all conditions for any given cell type to prevent analysis biases. Since ActD-treated LNCaP cells, as well as 22Rv1 cells in most conditions, tended to form clusters preventing separation of most nuclei, analyzed cells represented mostly periphery of the clusters or cells between clusters. We then computed for each Cajal body its mean signal intensity and diameter. In each sample, 800–1000 cells were analyzed.

### Phenotypic single cell analysis

Nucleus segmentation was done using StarDist [20], which localizes cell nuclei as star-convex polygons by training a convolutional neural network for predicting a polygon for the cell nucleus instance at each pixel location. We applied a pre-trained StarDist model (‘Versatile’) without transfer learning to the study data. For segmentation of subcellular organelles, methods robust towards intensity variations and background fluorescence, yet capable of small structures with varying appearance are needed. Several alternatives based on spatially enhancing filtering exist [19], and we chose to use Difference of Gaussians (DoG) filtering followed by thresholding both for segmentation of spots from what signal was the red (Coilin, 594) and the green (NPM, 488) channel. The parameters for filtering were experimentally set to optimize the trade-off between accurate spot representation and false positive detections. Feature extraction from nuclear and spot areas was done for the original intensity values within the segmentation masks and based on the segmentation masks for the object-based features. For visualization, we use violin plots [2, 9] and t-Distributed Stochastic Neighbor Embedding (t-SNE) [23] with Mahalanobis distance function and seeded random number generation for reproducibility and comparability.

### Statistical analysis

In cell density assay, two-way ANOVA was used to assess difference between treatment conditions, and unpaired t-tests were used to assess significance of differences between treatment conditions in other analyses using GraphPad Prism 6 software (GraphPad Software Inc., San Diego, CA, USA). A value of p ≤ 0.05 was statistically significant (p ≤ 0.05*, p ≤ 0.01**, p ≤ 0.001***).

## Results

### Cellular response of prostate cancer cells to platinum-based drugs

**Fig. 1:**
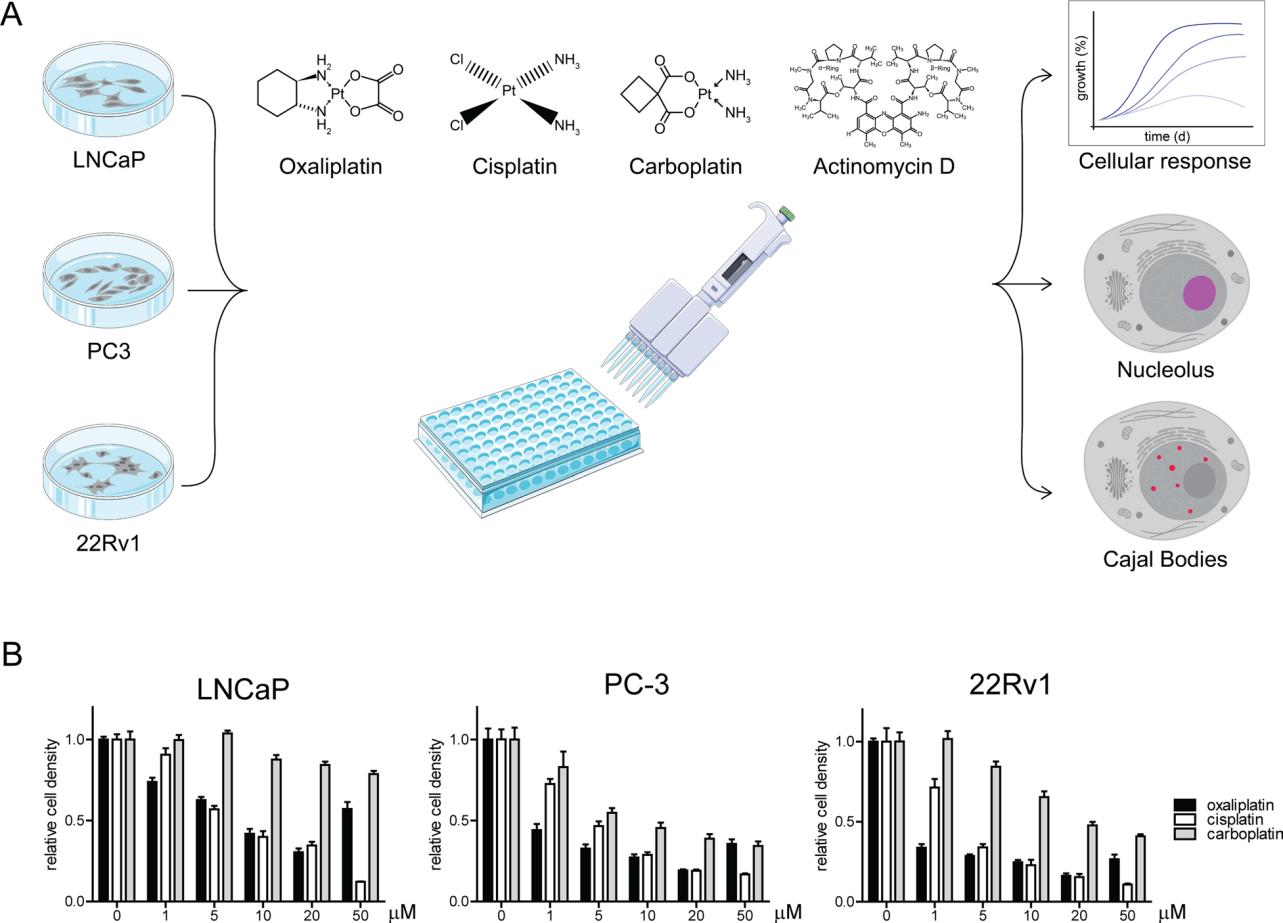
Assessing prostate cancer cell responses to platinum-based cytotoxic drugs. **A** Outline of the study. Three prostate cancer cell lines (LNCaP, PC-3, 22Rv1) are subjected to platinum-based cancer drugs (oxaliplatin, cisplatin, carboplatin) or Actinomycin D to assess their survival and the nuclear stress responses occurring in the nucleolus and Cajal bodies. **B** Relative cell density of the indicated cell lines measured to determine concentrations of platinum drugs inducing equal cytotoxicity. Cells were treated with the indicated concentrations of oxaliplatin, cisplatin and carboplatin for 96 h. Relative cell density is shown. Error bars, SEM

To assess responses of prostate cancer cells to platinum-based cytotoxic drugs, we utilized three cell lines with different AR status. LNCaP cells are AR positive, PC-3 cells AR negative, and 22Rv1 express a transcriptional variant AR-V7 in addition to full length AR. The cells were studied for their survival and markers for nucleoli and CBs to assess the nuclear cell responses to oxaliplatin, cisplatin, and carboplatin (Fig. 1A). We used Actinomycin D (ActD) as a positive control to induce nucleolar stress, as it has been well shown to induce RNA polymerase inhibition and NPM translocation from nucleoli (for a review, see [25] We first determined similarly effective doses of oxaliplatin, cisplatin, and carboplatin on the different cell lines by following cell density upon drug exposure between 1 and 50 μM concentrations (Fig. 1B). The results show that prostate cancer cells have different sensitivities to the platinum drugs tested (Fig. 1B), as LNCaP cells were least sensitive to carboplatin, while 22Rv1 cells were the most sensitive to cisplatin and oxaliplatin. Differences between the treatments were statistically significant (p < 0.001) in all cell lines between carboplatin and either cisplatin and oxaliplatin at 5–50 μM concentrations, as the differences between cisplatin and oxaliplatin were significant at the highest doses (50 μM; p < 0.001 for LNCaP, p < 0.05 for PC-3 and 22Rv1). Based on these results, the concentrations of 5 μM of oxaliplatin and cisplatin were used in the following experiments, while carboplatin was used at 50 μM for LNCaP, 5 μM of for PC-3, and 10 μM for 22Rv1 cells.

### Nucleolar stress response upon platinum treatment in prostate cancer cells

**Fig. 2:**
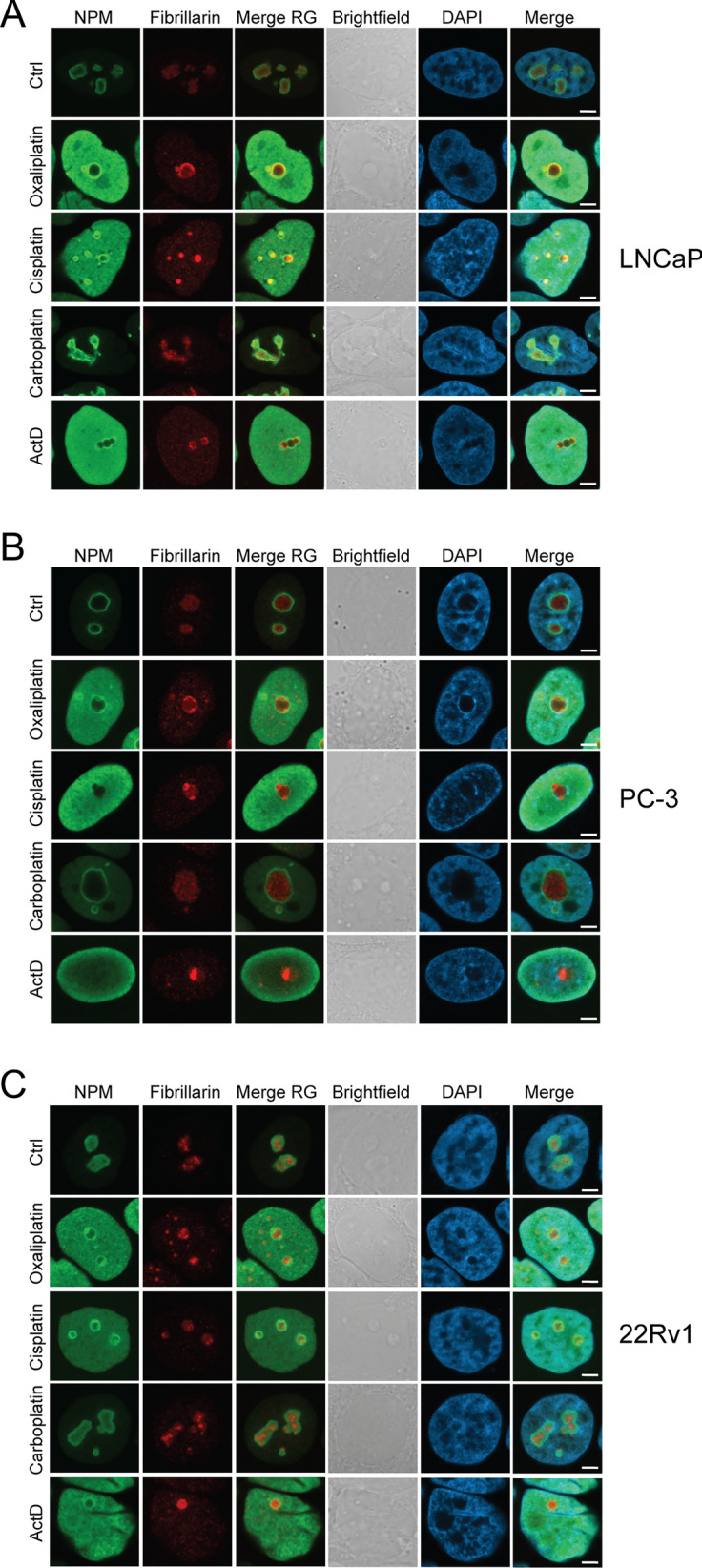
Nucleolar stress response in prostate cancer cells upon platinum drug treatments. Immunofluorescence staining of NPM and Fibrillarin showing the response of nucleoli after 24 h drug treatments in the nuclei of **A** LNCaP, **B** PC-3, and **C** 22Rv1 cells. NPM (green), Fibrillarin (red), merge of NPM and Fibrillarin (Merge RG), brightfield, DAPI, and Merge of NPM, Fibrillarin and DAPI is shown. Scale bars, 5 µm

We next determined the extent of nucleolar stress response following platinum-based drugs in LNCaP, PC-3, and 22Rv1 prostate cancer cells by immunofluorescence staining of nucleolar patterns of NPM and Fibrillarin as markers for granular and fibrillar components of the nucleoli, respectively. All three cell lines showed evidence of prominent nucleolar stress responses upon ActD as expected (Fig. 2, Additional file 1: Fig. 1). Of the platinum-based drugs, carboplatin was the least effective and oxaliplatin the most effective in inducing nucleolar stress in prostate cancer cells as measured with NPM translocation to nucleoplasm and Fibrillarin re-distribution to nucleolar caps. Cisplatin induced a prominent nucleolar stress response in PC-3 cells, a very mild response in 22Rv1 cells, and, interestingly, a heterogenous response in LNCaP cells (Fig. 2, Additional file 1: Fig. 1). The results show that while all the three cell lines have functional nucleolar stress response mechanisms, the extent of the reactions and level of heterogeneity between individual cells in response to platinum drugs varies.

### CB response to platinum treatment in prostate cancer cells

**Fig. 3:**
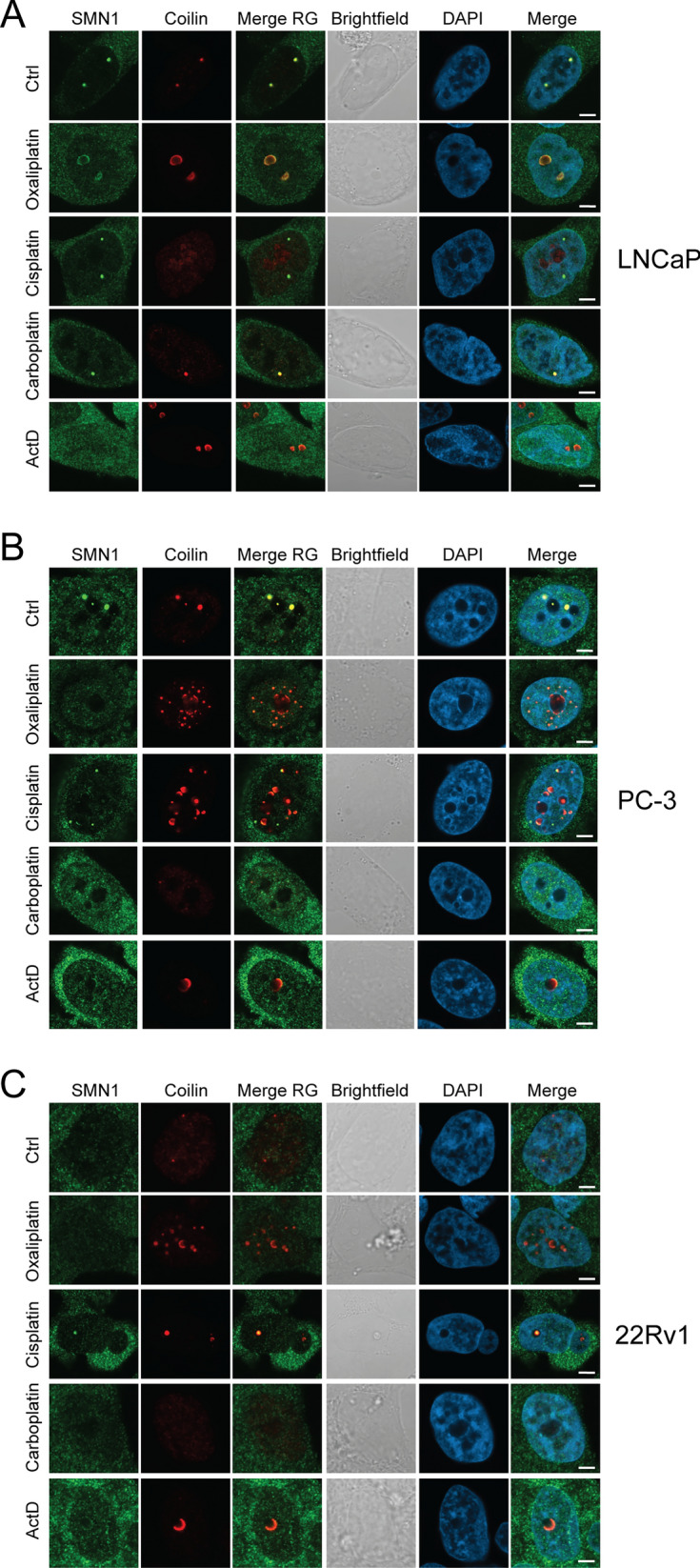
Cajal body stress response in prostate cancer cells upon platinum drug treatments. Immunofluorescence staining of SMN1 and Coilin showing the response of CBs after 24 h drug treatments in the nuclei of **A** LNCaP, **B** PC-3, and **C** 22Rv1 cells. SMN1 (green), Coilin (red), merge of SMN1 and Coilin (Merge RG), brightfield, DAPI, and Merge of SMN1, Coilin and DAPI is shown. Scale bars, 5 µm

Next, we tested whether platinum drugs induce CB stress in prostate cancer cells. By immunofluorescence staining, we determined the patterns of CBs based on two markers SMN1 and Coilin upon platinum drug or ActD-induced stress. Under control conditions, most LNCaP and PC-3 cells exhibit 1–2 CBs detectable with both Coilin and SMN1 (Fig. 3, Additional file 1: Figure S2). CBs are often localized near nucleoli in these cells. While Coilin localization is specific to CB structures in these cell lines, SMN1 can be readily detected also in the cytoplasm of all the three cell lines. Interestingly, only a minority of 22Rv1 cells have a clear CB as detected with the markers in question. Furthermore, the overall Coilin intensity is very low in 22Rv1 cells, while SMN1 staining is normally detected in the cytoplasm. Nucleolar stress induced by ActD results in loss of CBs, dispersal of nuclear SMN1 to nucleoplasm, and translocation of Coilin to chromatin-void nucleolar areas in all the cell lines. In general, platinum-based drugs induced a significant re-organization of CBs as measured with Coilin and SMN1 localization patterns. In most of these conditions, a decrease or loss of clear spots of nuclear SMN1 is visible in the majority of treated cells, while concentrated Coilin signals were detectable in most. Yet, co-localization of these proteins was still often detected. This indicates a more proficient dispersal of SMN1 from Cajal bodies during platinum-induced stress. More specifically, oxaliplatin induces an increase in number Coilin-positive spots in both LNCaP and PC-3 cells. Interestingly, oxaliplatin induces appearance of dotted pattern in Coilin staining also in 22Rv1 cells, indicating possible CB formation or at least similar Coilin stress response structures than in LNCaP and PC-3 cells, despite differences in steady-state CB levels. However, this de novo dotted pattern is not detected in the SMN1 staining. In cisplatin treated LNCaP and PC-3 cells, marked separation of nuclear Coilin and SMN1 staining occurred, with Coilin often localizing to chromatin devoid areas. Interestingly, in 22Rv1 cells quite the opposite occurred with a minority of cells with appearance of dotted co-localization of Coilin and SMN. In most 22Rv1 cells, Coilin localization in chromatin void areas is seen without co-localization of SMN. Carboplatin did not induce marked differences in CB marker localization in any of the prostate cancer cells tested, although decreased signal and spot intensities occurred by visual detection (Fig. 3, Additional file 1: Figure S2, Additional file 2: Table S1).

**Fig. 4:**
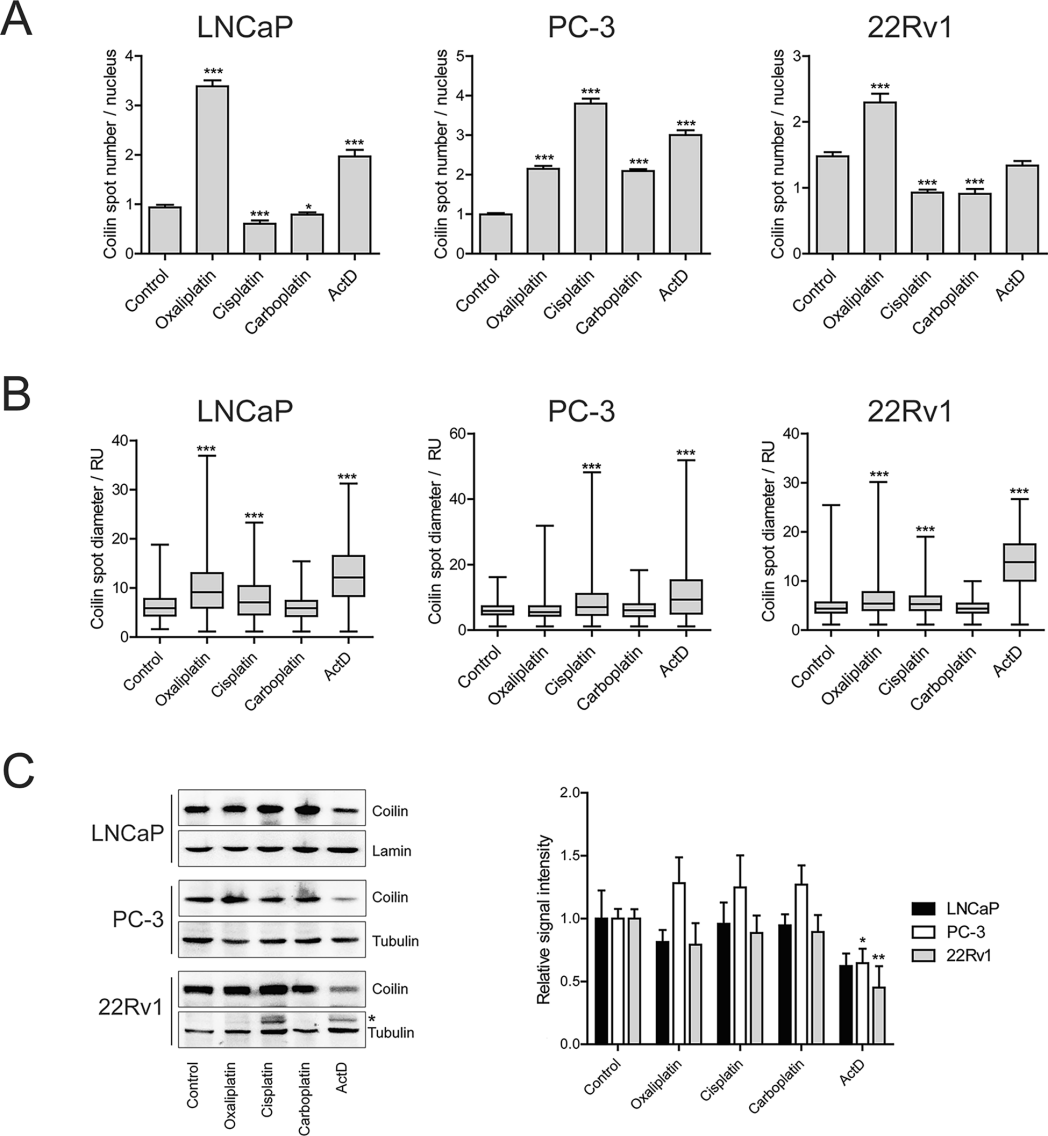
Quantitative assessment of Coilin as a Cajal body marker in platinum drug responses in prostate cancer cells. **A** Numbers of CBs in nuclei of the indicated cell lines treated by the indicated drugs as measured with Coilin dots. **B** Size of CBs of the indicated cell lines treated by the indicated drugs as measured with Coilin spot diameter. **C** Western blot analysis of Coilin protein levels in the indicated cell lines treated by platinum drugs. Left panel, representative blot images. Tubulin and lamin shown as loading controls, non-specific bands indicated with an asterisk. Right panel, relative signal intensity for three replicate experiments for each cell line. Mean values with S.D., *p-value < 0.05, **p-value < 0.01

Since CBs underwent clear alterations upon platinum drug treatment in prostate cancer cells as measured via Coilin and SMN1 localization, we quantified this phenomenon using Coilin as a marker. Quantitation of the number and size of Coilin-positive spots per nuclei verified that oxaliplatin increased the spot number significantly in all cell lines (Fig. 4A). In LNCaP and 22Rv1 cells, oxaliplatin also led to an increase in spot size as measured based on Coilin spot diameter (Fig. 4B). In contrast in PC-3 cells, Coilin spot diameter did not change significantly. In cisplatin- and carboplatin-treated cells, Coilin spot number increased in PC-3 cells but decreased in LNCaP and 22Rv1 cells. An increase in Coilin spot diameter was detected in all the cell lines upon cisplatin treatment, while carboplatin did not influence the spot size in any of the cell lines. Most of these changes occurred without major alterations in overall Coilin protein levels (Fig. 4C), despite that Coilin expression was readily reactive to drug-induced stress in all the cell lines as demonstrated by ActD treatment, and that a trend towards increased Coilin levels upon platinum drugs was detected in PC-3 cells. Taken together, these results show that a stress response of CBs follows exposure of prostate cancer cells to platinum drugs, and this is quantitatively reflected in Coilin immunostaining patterns.

### Specific and heterologous nuclear responses to platinum drugs in prostate cancer cells

**Fig. 5:**
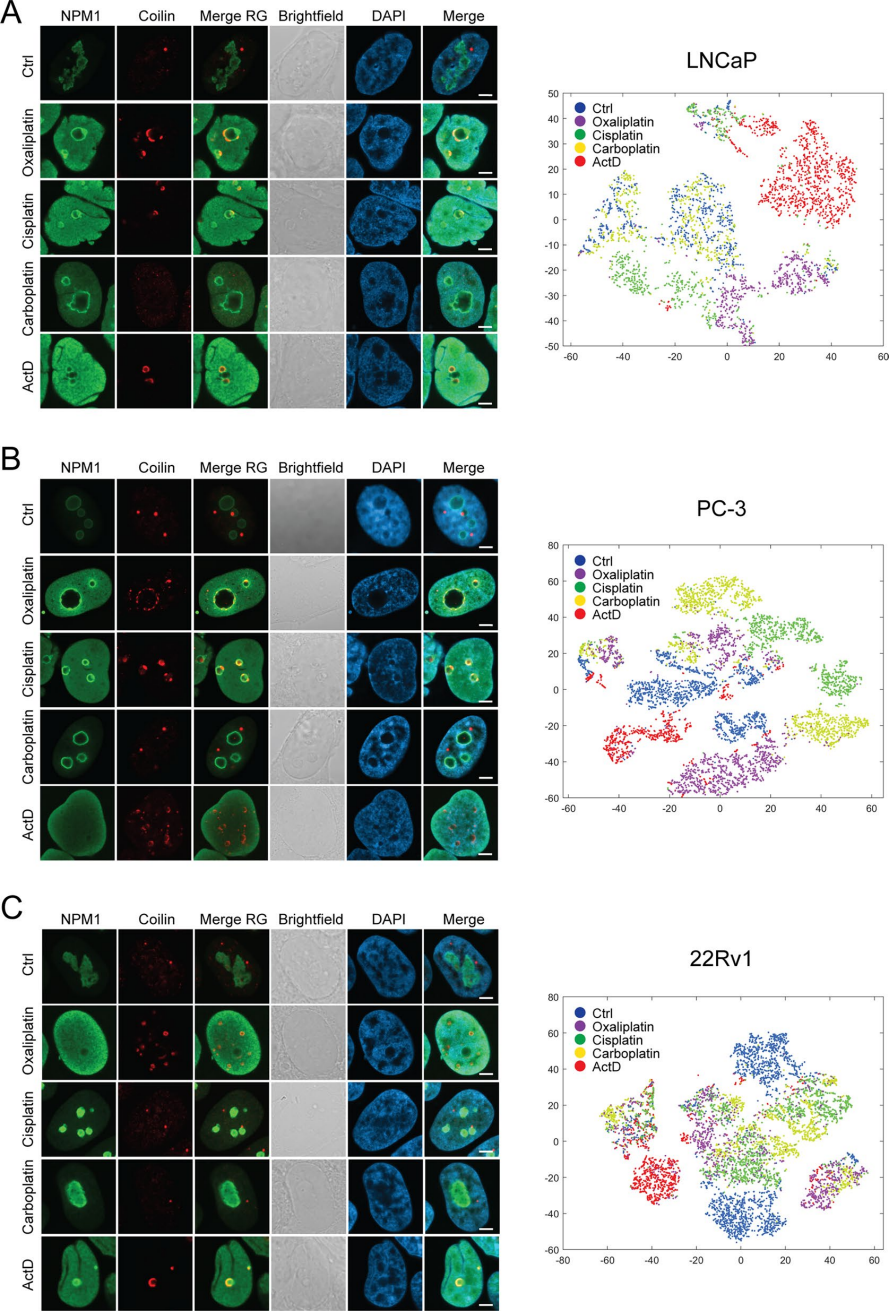
Phenotypic assessment of prostate cancer cell nuclear stress responses upon platinum drug treatments. NPM and Coilin immunofluorescence staining -based quantitative analysis of the phenotypes of **A** LNCaP, **B** PC-3, and **C** 22Rv1 cells in response 24 h drug treatments. Scale bars, 5 µm. Left panels: example nuclei in the immunofluorescence staining. NPM (green), Fibrillarin (red), merge of NPM and Fibrillarin (Merge RG), brightfield, DAPI, and Merge of NPM, Fibrillarin and DAPI is shown. Right panels: t-sne of computational feature-based single-cell analysis showing phenotypic distributions of the drug responses in each cell line

Since Coilin was observed to localize to chromatin void areas upon platinum drug-induced stress and the number of Coilin spots seemed to change, we tested whether Coilin translocates to nucleoli in prostate cancer cells in response to platinum drugs. Co-immunostaining of Coilin with nucleolar marker NPM showed that, as expected, CBs were clearly separate structures from the nucleoli under normal growth conditions in all cell lines tested (Fig. 5). In response to platinum drug treatments, prostate cancer cells exhibited highly heterologous changes in distribution of Coilin-immunopositivity in relation to nucleoli (Fig. 5) with frequent localization of Coilin close or within the nucleoli. There were differences between responses to the different drugs within and between the cell lines tested. Especially upon oxaliplatin and cisplatin treatments, heterogenous phenotypes within each cell line were detected. Hence, we classified the nuclear phenotypes based on combined NPM and Coilin staining as surrogate markers for nuclear and CB changes following cellular stress (Additional file 1: Figure S3). Nucleolar capping, nucleolar localization, nucleoplasmic diffusion, CB dispersion, or no stress-induced phenotype were evidenced. The results showed that while carboplatin induced nucleoplasmic diffusion of Coilin in a minority of 22Rv1 cells, the phenotype mostly remained normal in all cell lines. Cisplatin induced some capping and nucleolar localization of Coilin in all cell lines, most prominently in PC-3 cells. Oxaliplatin induced mostly Coilin capping in LNCaP cells, while in PC-3 and 22Rv1 also cells with nucleoplasmic diffusion and dispersal of CBs to multiple Coilin spots were evident.

Since markers of nucleoli and CBs revealed significant differences and heterogeneity in prostate cancer cells to cytotoxic drug treatment, we wanted to quantitatively analyze the similarity of drug-responsive cell populations within and between the prostate cancer cell lines. For this, we performed an image-based single cell phenotypic analysis of the nuclear stress responses of LNCaP, PC-3 and 22Rv1 cells following platinum drug or ActD treatments. Features of immunostained NPM and Coilin signals were analyzed and used to depict each cell in a t-sne plot for each cell line (Fig. 5, Additional file 1: Figure S4). The results show that under normal growth conditions, LNCaP and PC-3 cells are relatively homogenous as the control cells are situated relatively close together in the t-sne space. In 22Rv1 cells, control cells are separated in two major clusters, indicating two nuclear phenotypes in these cells under normal growth conditions. ActD induces a phenotype with mostly well separated, clear cluster in all the cell lines. It is interesting that while carboplatin affected the translocation of NPM and Coilin the least of the drugs tested, the phenotype of carboplatin is intermixed with control cells only in the LNCaP cell line. In PC-3 cell line, cells are separated into many clusters upon carboplatin, while in 22Rv1 cells the carboplatin clusters are intermixed with especially those of cisplatin. In contrast, in PC-3 cells cisplatin has a clearly separate nuclear stress phenotype, while in LNCaP cells it divides in two populations. Interestingly, oxaliplatin exhibits opposite behaviour, with a separate population in LNCaP cells but split clusters in PC-3 cells. In the 22Rv1 cells, the cell populations of all platinum drug treatments are most intermixed, indicating least amount of selective nuclear responses in this cell line (Fig. 5, Additional file 1: Figure S4).

### Effects of stress response proteins FUS and TDP-43 on nuclear platinum drug responses in prostate cancer cells

**Fig. 6:**
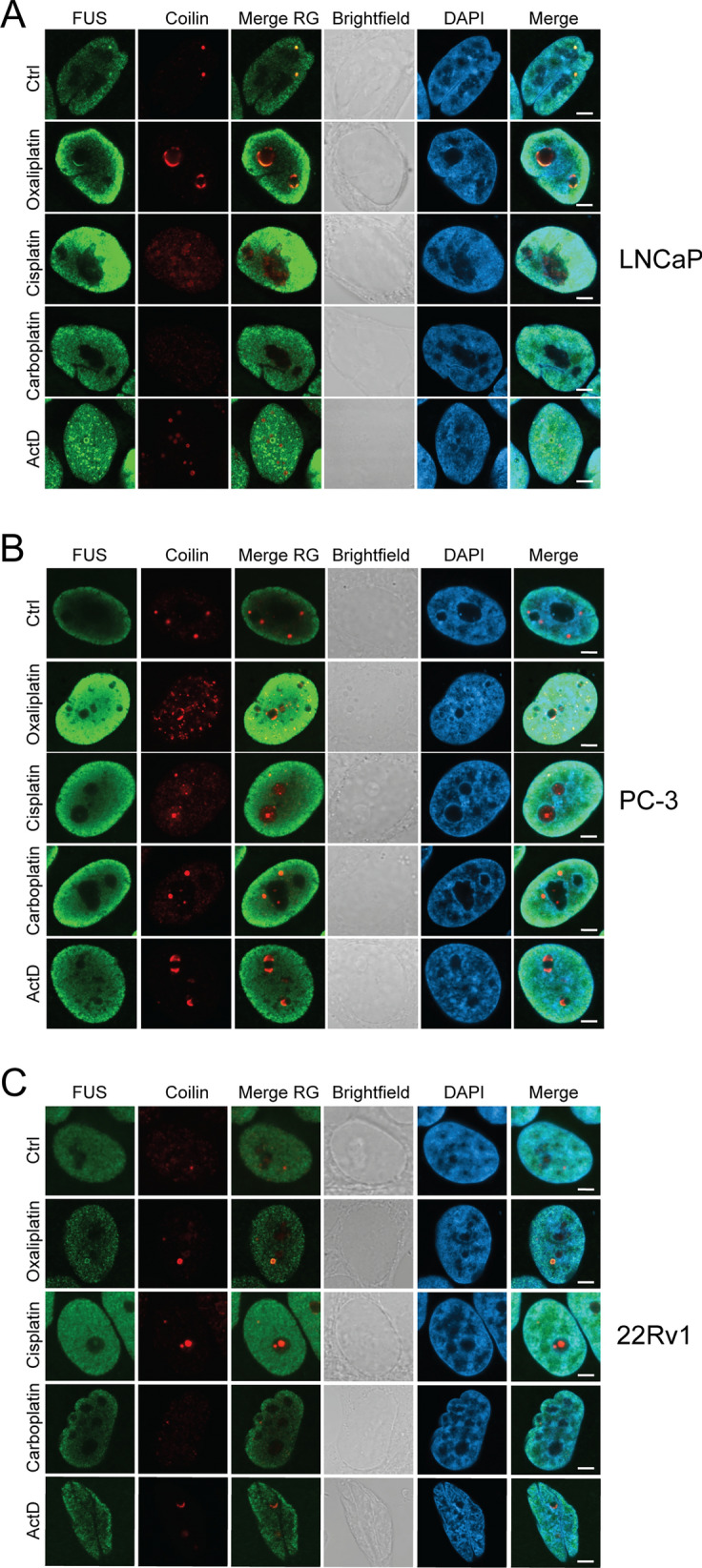
Localization of FUS in prostate cancer cells upon platinum drug treatments. Immunofluorescence staining of FUS and Coilin the localization response to 24 h drug treatments in the nuclei of **A** LNCaP, **B** PC-3, and **C** 22Rv1 cells. FUS (green), Coilin (red), merge of FUS and Coilin (Merge RG), brightfield, DAPI, and Merge of FUS, Coilin and DAPI is shown. Scale bars, 5 µm

**Fig. 7:**
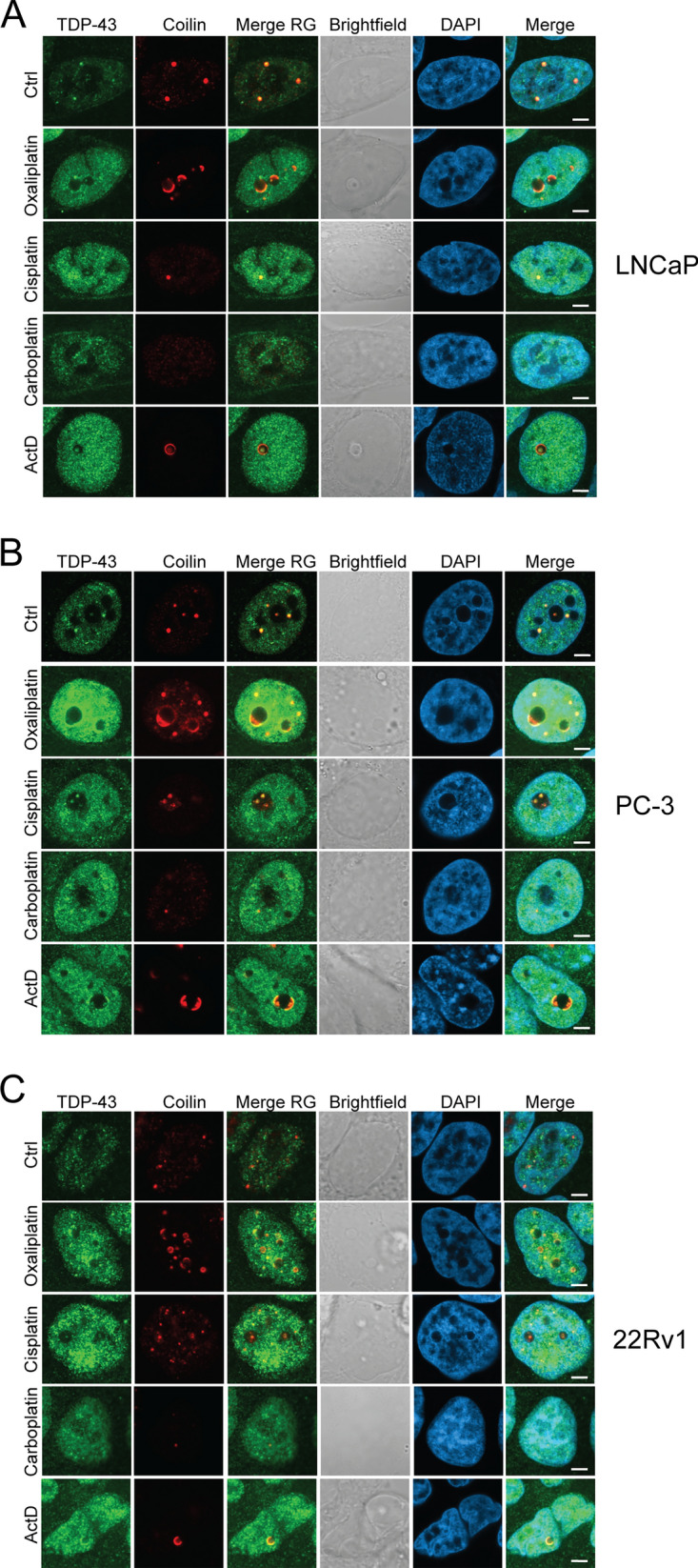
Localization of TDP-43 in prostate cancer cells upon platinum drug treatments. Immunofluorescence staining of TDP-43 and Coilin showing the localization response to 24 h drug treatments in the nuclei of A) LNCaP, B) PC-3, and C) 22Rv1 cells. TDP-43 (green), Coilin (red), merge of TDP-43 and Coilin (Merge RG), brightfield, DAPI, and Merge of TDP-43, Coilin and DAPI is shown. Scale bars, 5 µm

Since CBs underwent drastic changes upon platinum drug exposure as measured by translocation of two significant CB proteins Coilin and SMN1, we then asked what other proteins might be involved in CB homeostasis in prostate cancer cells. Two stress responsive factors, FUS and TDP-43, are previously indicated in CB formation and functions in neuronal models (Fakim and Vande Welde 2023). We first tested by immunostaining how FUS and TDP-43 respond to platinum-based drugs in prostate cancer cells. Co-immunostaining with Coilin revealed that FUS is nucleoplasmic and exhibits clear localization in CBs only in LNCaP cells of the prostate cancer cell lines tested (Fig. 6, Additional file 1: Figure S5). This co-localization is retained in carboplatin treated LNCaP cells, while occasional co-localization of FUS and Coilin is detected upon other platinum drug treatments in the prostate cancer cell lines tested. In contrast, while the majority of TDP-43 is nucleoplasmic, TDP-43 and Coilin overlap prominently in CBs under control conditions. Especially in LNCaP and PC-3 cells, all Coilin spots are occupied with TDP-43 (Fig. 7, Additional file 1: Figure S6). In 22Rv1 cells, the co-localization is less prominent, likely due to low levels of Coilin-intense CBs. Upon ActD, the co-localization is lost, and the nucleolar Coilin signal is separated from the TDP-43 that remains in the nucleoplasm, but co-localization occurs in the nucleolar periphery in nucleolar caps. Upon platinum drugs, TDP-43 signal levels in the nucleoplasmic area are increased. Carboplatin induces the mildest changes to TDP-43 localization and intensity, and co-localization with Coilin can be detected in spots in the nucleoplasm of some cells. In cisplatin and oxalipatin treated cells, co-localization of the proteins is often prominent in nucleoplasmic spots, and especially in oxaliplatin-treated cells TDP-43 often co-occupies nucleolar caps with Coilin (Fig. 7, Additional file 1: Figure S6).

**Fig. 8:**
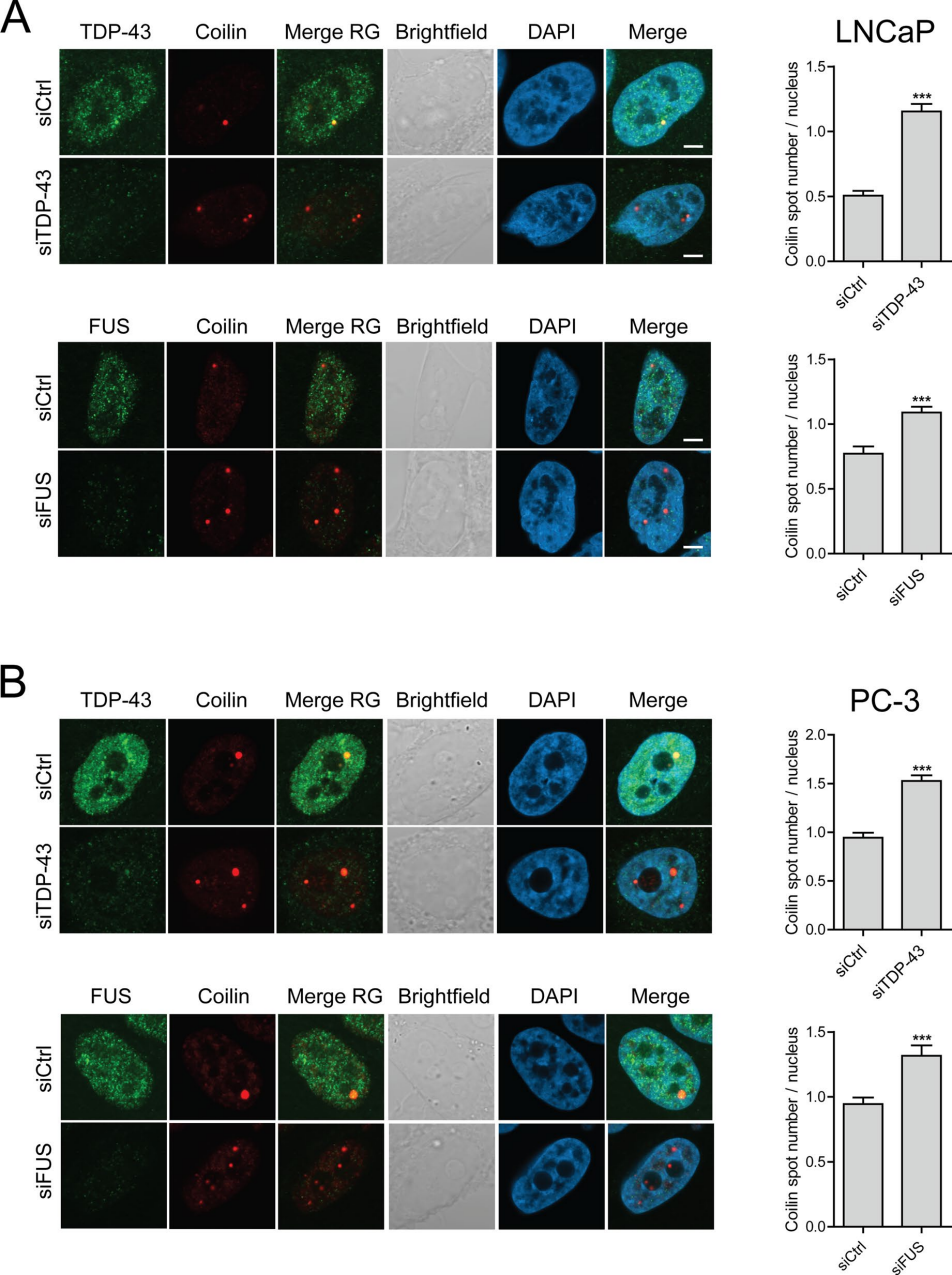
TDP-43 and FUS regulate Cajal body organization in prostate cancer cells. Knock-down of TDP-43 and FUS with siRNA targeting in LNCaP and PC-3 cells followed by immunofluorescence staining of TDP-43 or FUS and Coilin in the nuclei of the cells. Left panels: TDP-43 or FUS (green), Coilin (red), merge of TDP-43 or FUS with Coilin (Merge RG), brightfield, DAPI, and Merge of TDP-43 or FUS with Coilin and DAPI is shown. Scale bars, 5 µm. Right panels: Quantitation of CBs according to Coilin spot number in the siRNA-treated cells

The differences observed in FUS and TDP-43 localizations in platinum drug-treated cells are not due to changes in protein levels, as they remain constant for both proteins as opposed to a prominent decrease of TDP-43 upon ActD in all cell lines (Additional file 1: Figure S7). Hence, we then tested for functional significance of FUS and TDP-43 for CBs in prostate cancer cells. siRNA downregulation of either FUS or TDP-43 in LNCaP and PC-3 cells induced an increase in Coilin spot count suggestive of an increased number of CBs (Fig. 8). Similar effects were observed in 22Rv1 cells (Additional file 1: Figure S8). This was associated with increased cell growth under normal growth conditions (Additional file 1: Figure S9). The effect on CBs was more prominent with downregulation of TDP-43 than FUS in both cell lines (Fig. 8), and downregulation of TDP-43 was associated with a trend for increased survival in both cell lines upon carboplatin treatment (Additional file 1: Figure S9; p-value 0.059 for PC-3). In LNCaP and 22Rv1 cells, expression levels of Coilin were unaffected by downregulation of both FUS and TDP-43, while in PC-3 cells siTDP-43 but not siFUS induced downregulation of Coilin levels 72 h post-transfection (Additional file 1: Figure S7). These results associate TDP-43 with nuclear stress responses to platinum drugs and indicate a role for TDP-43 in CB regulation in prostate cancer cells.

## Discussion

Platinum compounds have moderate anti-tumour activity in patients with advanced prostate cancer, and it is important to find selection markers for prostate cancer patients that are most likely to benefit from platinum-based therapy [6, 18]. To make better informed decisions, understanding the responses in prostate cancer cells to the platinum drugs is crucial. Here, we show that advanced prostate cancer cells show heterologous responses to different platinum drugs, and that nuclear stress responses to the different drugs vary. We identify that, in addition to nucleolar stress responses, platinum drugs induce a prominent CB stress response.

We studied the phenotype of CBs and their response to platinum-based cytotoxic drugs in prostate cancer cells and found significant reorganization of these organelles following the treatments. Here, we show that cisplatin and oxaliplatin induce a prominent translocation response of Coilin associated with dispersal of CBs. On the other hand, carboplatin does not induce such significant alterations in Coilin localization under the concentrations used that promote similar cytotoxic effects in these cells. The different potency in inducing a CB stress response may contribute to the effectiveness of these drugs against different types of cancers.

CBs function in RNA processing, and Coilin has a role in snRNP biogenesis. Coilin has previously been shown to participate in downregulation of RNA polymerase I activity upon cisplatin-induced DNA damage [6]. While Coilin is detected and used here as a surrogate marker for quantitation of stress-induced CB alterations, the Coilin-positive nuclear spots do not necessarily always represent functional CBs. Hence, it is important in future studies to determine the molecular composition and functionality of the stress-induced structures and their relationship to snRNP regulation during cancer cell responses to drug treatments.

The differentiating nuclear stress responses to the different drugs may be related to the type and extent of the DNA damage inflicted on the cells. Even though platinum-based drugs induce the canonical DDR, evidence is accumulating for a role of inhibition of RNA polymerase I and ribosome biogenesis [10, 22]. We showed here that, in addition to the previously reported ribosomal and nucleolar stress response induced by oxaliplatin, a prominent CB stress response is also induced by this drug. This effect is detectable also with cisplatin. However, our results indicate that carboplatin does not induce a marked CB stress response in prostate cancer cell lines at comparable cytotoxic doses than cisplatin and oxaliplatin. Whether this is the case also in healthy prostate epithelial cells, and in cells of other cancer types, remains to be investigated.

Our results show that while prostate cancer cells have functional nucleolar stress response mechanisms, the extent of the reactions vary and are not in direct relationship with survival of the cells. For example, PC-3 cells were most sensitive to the cytotoxic effects of carboplatin, yet they did not exhibit a prominent nucleolar stress response upon that drug. PC-3 cells also exhibited unique increase in CB number upon cisplatin and carboplatin treatment, indicating differential CB stress response than the other, AR-positive cell lines studied. While our experiments did not assess the role of AR in the platinum drug responses of these cell lines, it is interesting to speculate that the exceptional stress response phenotypes of PC-3 cells could be associated with loss of AR. Furthermore, 22Rv1 expressing the AR-V7 transcript variant have hardly a detectable CBs under control conditions. Yet, oxaliplatin induces appearance of dotted pattern in Coilin staining also in these cells, indicating possible CB formation or at least similar Coilin stress response than in the other cell lines, despite differences in steady-state CB levels. These results suggest that CBs may contribute to the heterogeneity in prostate cancer and affect the success of the use of platinum compounds.

FUS and TDP-43 are RNA binding proteins that contribute to CB formation [5]. We showed here that both proteins contribute to CB organization also in prostate cancer cells. TDP-43 was more prominent to induce dispersal of Coilin than FUS when downregulated, and TDP-43 exhibited clear stress-induced localization changes and associations with Coilin upon platinum drugs. We found that TDP-43 co-localization with Coilin is lost upon carboplatin treatment, and downregulation of TDP-43 indicated modest survival benefit under those conditions. This suggests that dissociation of TDP-43 from Coilin-positive structures promotes its functions in anti-survival pathways. The role of TDP-43 and co-regulation of TDP-43 and Coilin and/or CBs warrants further investigation in terms of cancer drug responses especially since TDP-43 levels decrease in advanced prostate cancer [11] and this may affect the nuclear responses of prostate cancer cells to cytotoxic drugs.

Since markers of nucleoli and CBs revealed significant differences and heterogeneity in prostate cancer cells to cytotoxic drug treatments, we quantitatively analyzed the similarity of drug-responsive cell populations within and between the prostate cancer cell lines. Our image-based single cell phenotypic analysis combines markers of nucleolar and CB stress responses to depict the heterogeneity in nuclear reactions to drugs, allowing dissection of heterogeneity of drug responses at a single cell level. This and similar tools can be useful in determining the populations of cancer cells evading cytotoxicity, gaining drug resistance, and enabling re-growth of resistant populations. Identification of the stress response phenotypes of such populations can reveal key drug responsive pathways to target in attempts to fight drug resistance and relapse in patients.

### Supplementary Information

**Additional file 1: Figure S1.** Nucleolar stress response in prostate cancer cells upon platinum drug treatments. **Figure S2.** Cajal body stress response in prostate cancer cells upon platinum drug treatments. Quantitation of nuclear stress phenotypes in prostate cancer cells upon platinum drug treatments. **Figure S3.** Quantitation of nuclear stress phenotypes in prostate cancer cells upon platinum drug treatments. **Figure S4.** Feature-based single-cell phenotypic analysis of nuclear stress phenotypes in prostate cancer cells upon platinum drug treatments. **Figure S5.** Localization of FUS in prostate cancer cells upon platinum drug treatments. **Figure S6.** Localization of TDP-43 in prostate cancer cells upon platinum drug treatments. **Figure S7.** Response of TDP-43 and FUS to platinum drugs in prostate cancer cells. **Figure S8.** Effect of TDP-43 and FUS in Coilin spots in 22Rv1 cells. **Figure S9.** Role of TDP-43 and FUS in platinum drug responses and regulation of Coilin in prostate cancer cells

**Additional file 2: Table S1.** Predominant nuclear signals of Cajal body proteins SMN1 and Coilin in cells treated with the indicated drugs for 24 h. CB, Cajal bodies.

## Data Availability

The data that support the findings of this study are available from the corresponding author upon reasonable request.
