# Supplementary material for: Platinum-based drugs induce phenotypic alterations in nucleoli and Cajal bodies in prostate cancer cells
Source: Cancer Cell Int. 2024 Jan 13;24:29. doi: 10.1186/s12935-023-03205-0 (PMC10790272; doi:10.1186/s12935-023-03205-0)
Supplement: Supplementary file 1 — Additional file 1: Figure S1. Nucleolar stress response in prostate cancer cells upon platinum drug treatments. Figure S2. Cajal body stress response in prostate cancer cells upon platinum drug treatments. Quantitation of nuclear stress phenotypes in prostate cancer cells upon platinum drug treatments. Figure S3. Quantitation of nuclear stress phenotypes in prostate cancer cells upon platinum drug treatments. Figure S4. Feature-based single-cell phenotypic analysis of nuclear stress phenotypes in prostate cancer cells upon platinum drug treatments. Figure S5. Localization of FUS in prostate cancer cells upon platinum drug treatments. Figure S6. Localization of TDP-43 in prostate cancer cells upon platinum drug treatments. Figure S7. Response of TDP-43 and FUS to platinum drugs in prostate cancer cells. Figure S8. Effect of TDP-43 and FUS in Coilin spots in 22Rv1 cells. Figure S9. Role of TDP-43 and FUS in platinum drug responses and regulation of Coilin in prostate cancer cells [file 12935_2023_3205_MOESM1_ESM.pdf]

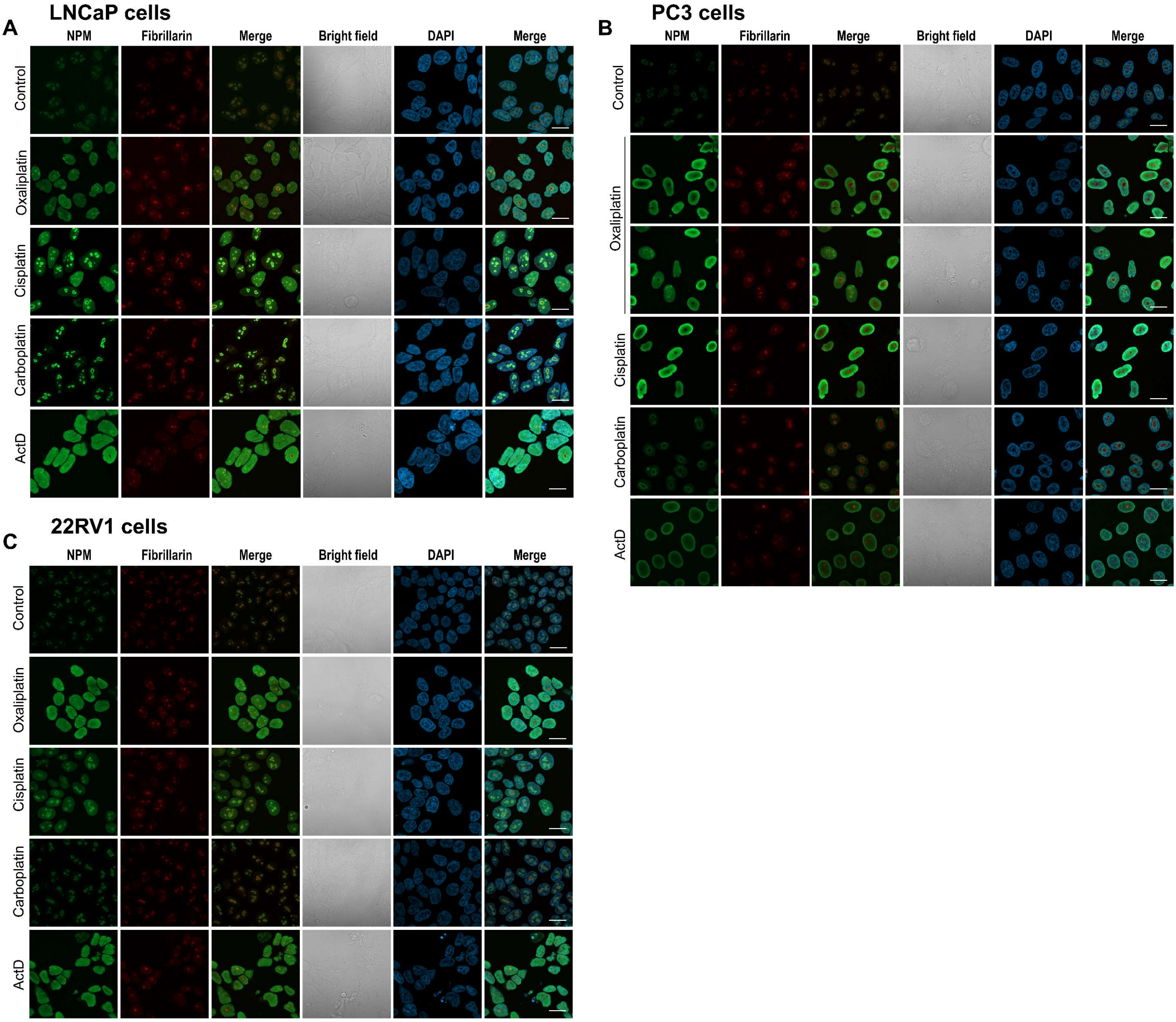

Additional file 1: Figure S1. Nucleolar stress response in prostate cancer cells upon platinum drug treatments. Immunofluorescence staining of NPM and Fibrillarin showing the response after 24 hrs drug treatments in A) LNCaP, B) PC-3, and C) 22Rv1 cells. NPM (green), Fibrillarin (red), merge of NPM and Fibrillarin (Merge RG), brightfield, DAPI, and Merge of NPM, Fibrillarin and DAPI is shown. Scale bar, 20 μm. Related to Figure 2.

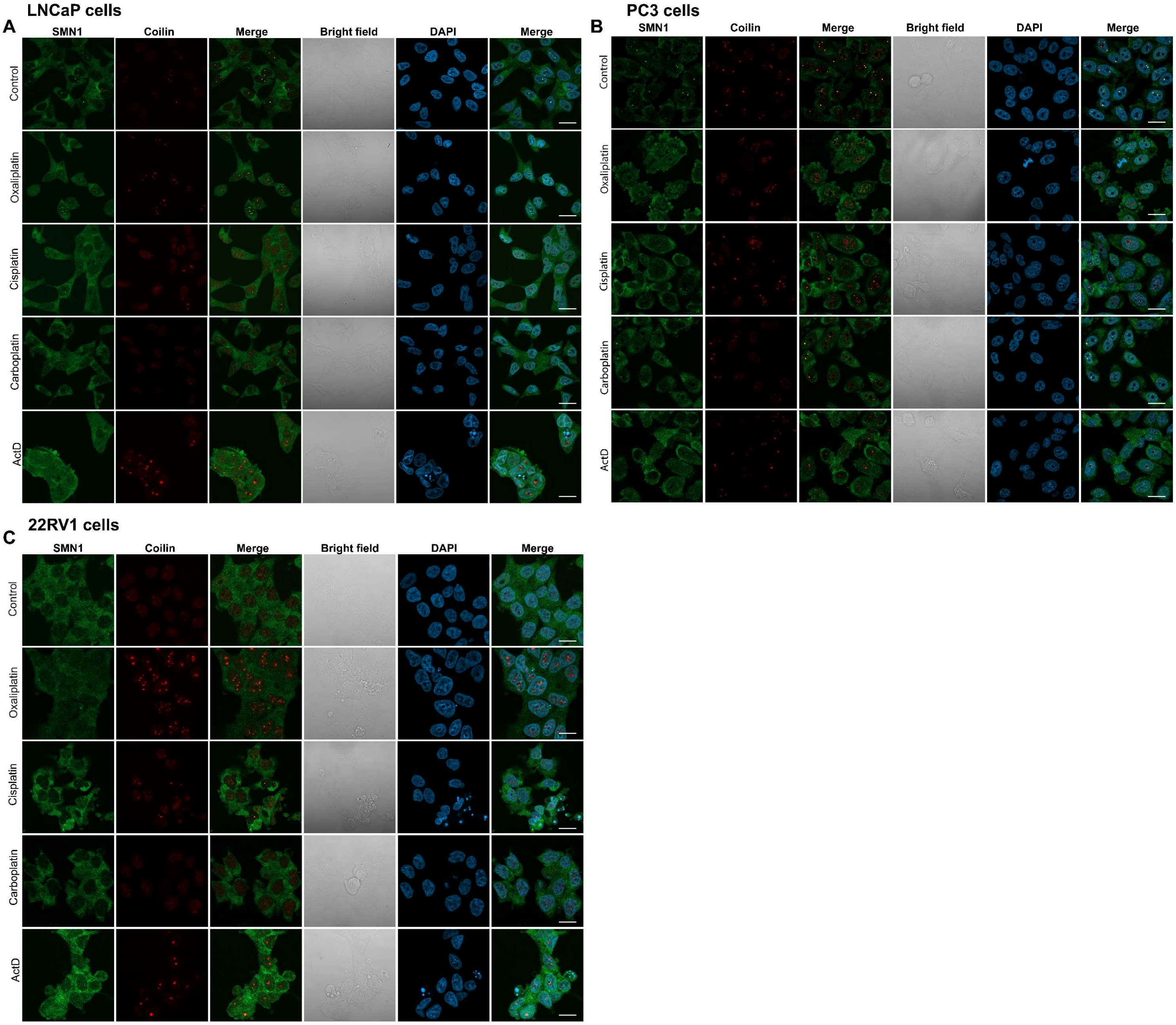

Additional file 1: Figure S2. Cajal body stress response in prostate cancer cells upon platinum drug treatments. Immunofluorescence staining of SMN1 and Coilin showing the response of CBs after 24 hrs drug treatments in A) LNCaP, B) PC-3, and C) 22Rv1 cells. SMN1 (green), Coilin (red), merge of SMN1 and Coilin (Merge RG), brightfield, DAPI, and Merge of SMN1, Coilin and DAPI is shown. Scale bar, 20 μm. Related to Figure 3.

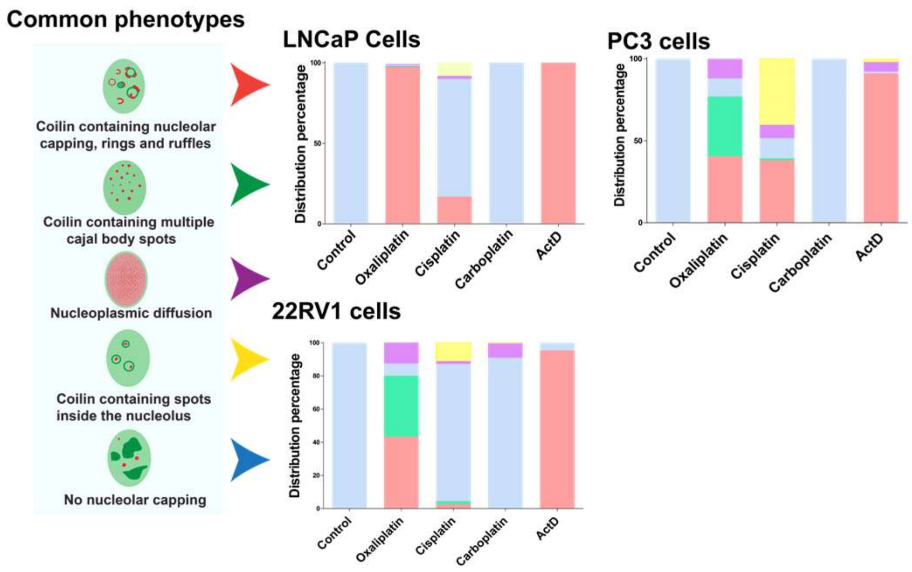

Additional file 1: Figure S3 Quantitation of nuclear stress phenotypes in prostate cancer cells upon platinum drug treatments. Each cell was categorized to one of the five indicated categories based on immunofluorescence staining of NPM and Coilin.

LNCaP

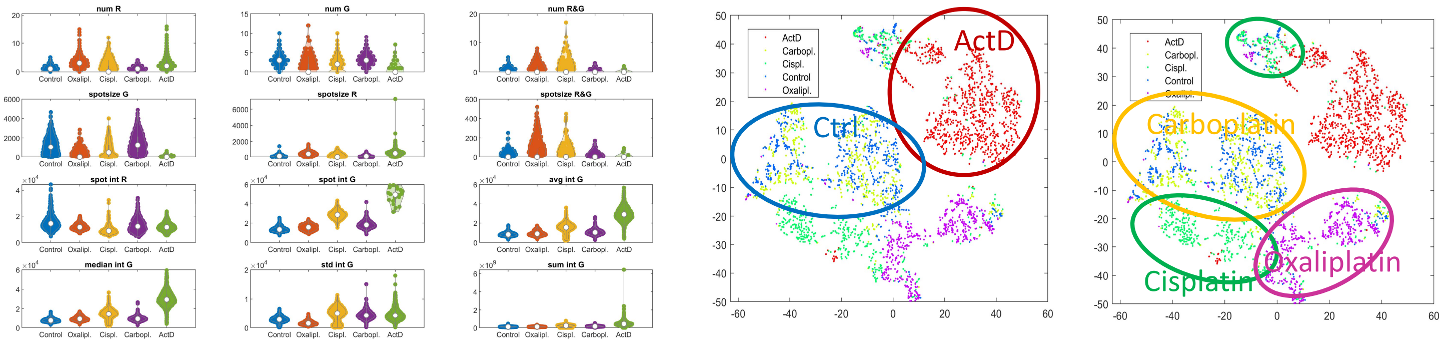

PC-3

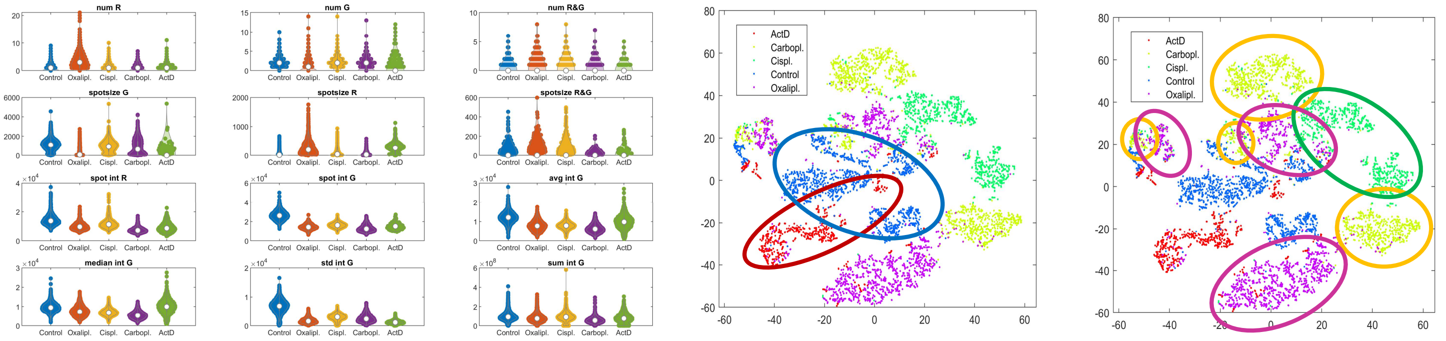

22Rv1

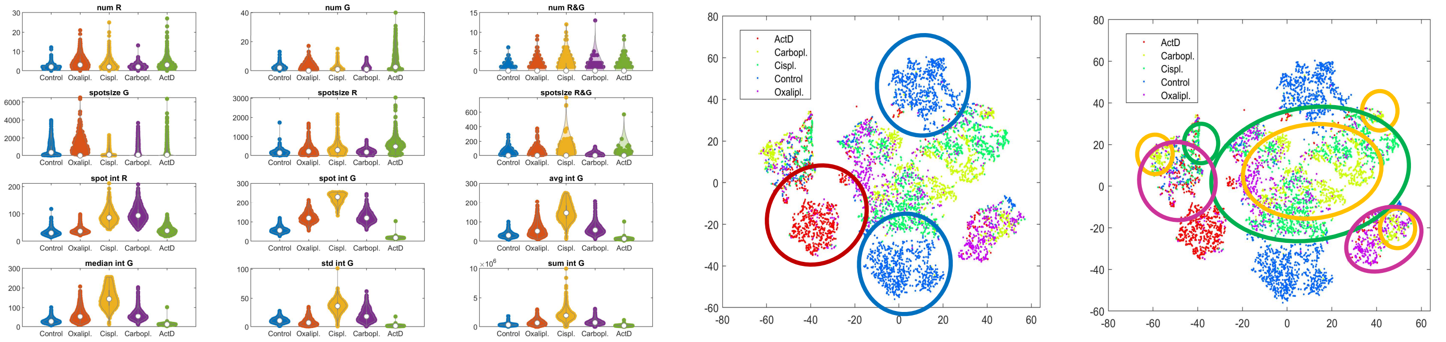

Additional file 1: Figure S4. Feature-based single-cell phenotypic analysis of nuclear stress phenotypes in prostate cancer cells upon platinum drug treatments. Computational analysis based on immunofluorescence staining of NPM and Coilin showing phenotypic contributions of the drug responses in each cell line. Left panels, violin plots of individual feature values used in the analysis. Right and middle panels: the same t-sne figures as in Figure 5, with circles indicating clustersinduced by of each drug. Features used were as follows: num R = number of red spots per each cell (G = green, R&G = overlapping): size G = size of green spots (in pixels) in a cell; avg int G = mean green intensity in nucleus area; median int G = median green intensity in nucleus area; std int G = standard deviation of green intensity in nucleus area; sum int G = sum of green intensity in nucleus area. Related to Figure 5.

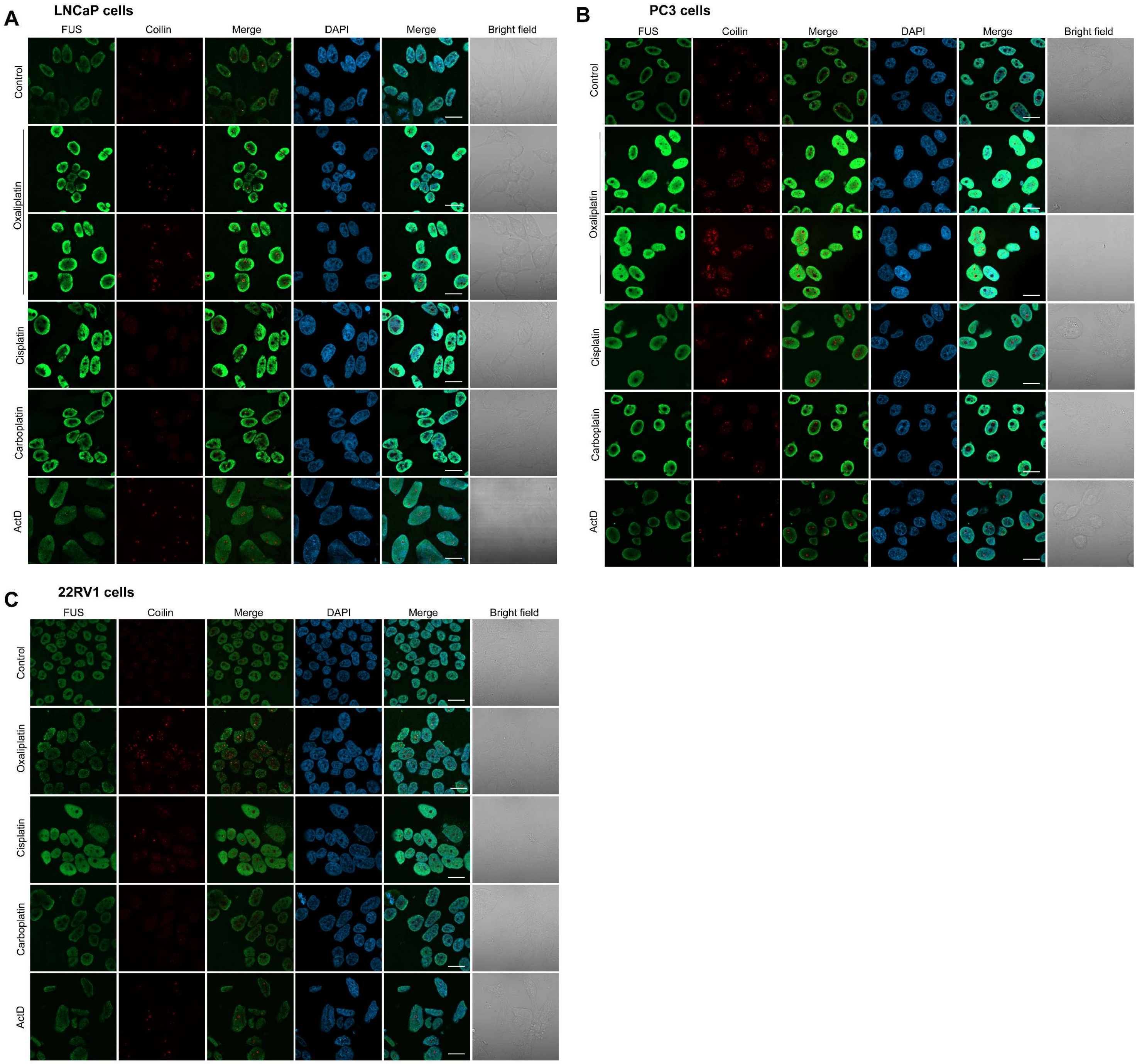

Additional file 1: Figure S5. Localization of FUS in prostate cancer cells upon platinum drug treatments. Immunofluorescence staining of FUS and Coilin the localization response to 24 hrs drug treatments in A) LNCaP, B) PC-3, and C) 22Rv1 cells. FUS (green), Coilin (red), merge of FUS and Coilin (Merge RG), brightfield, DAPI, and Merge of FUS, Coilin and DAPI is shown. Scale bar, 20 μm. Related to Figure 6.

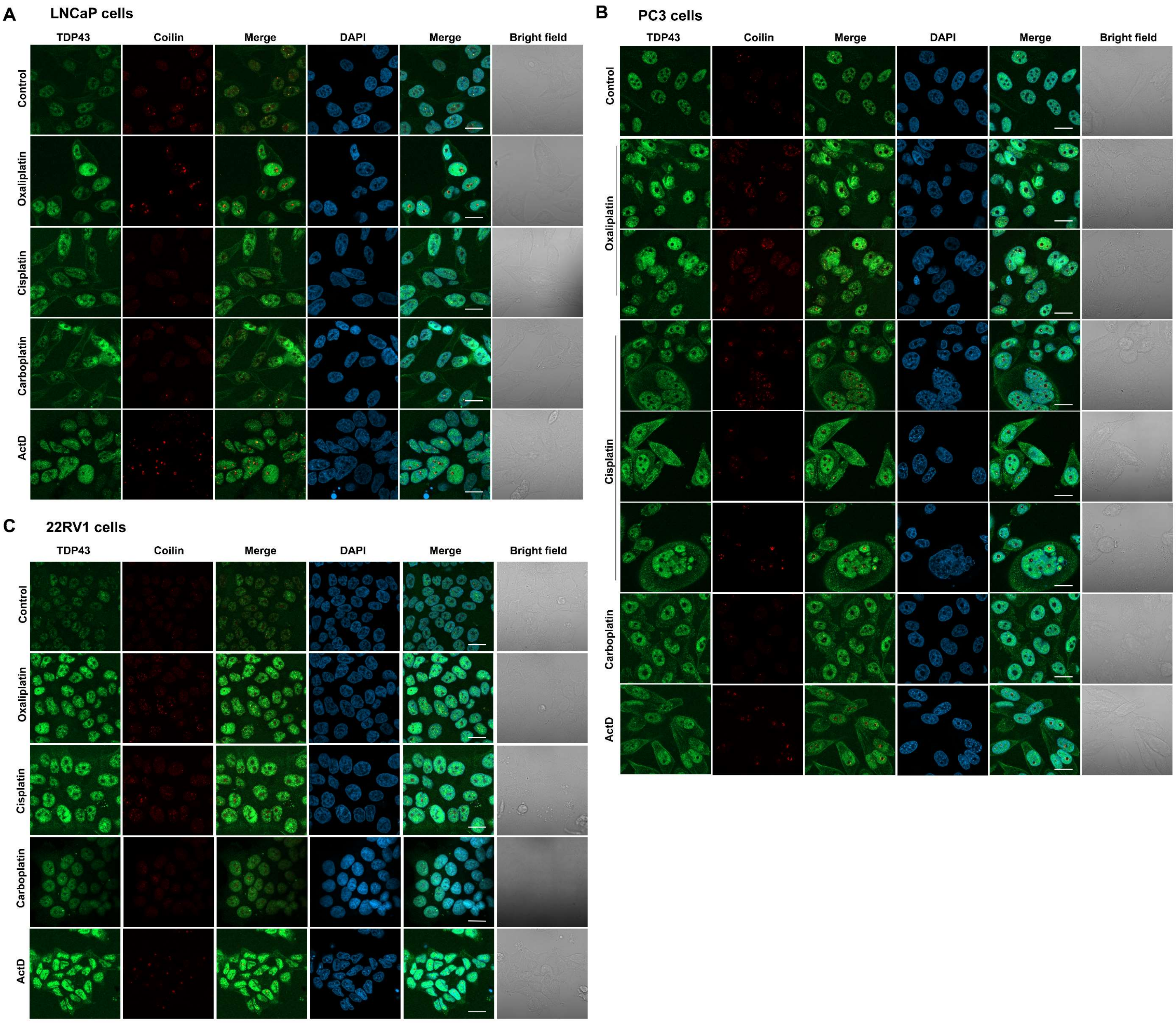

Additional file 1: Figure S6. Localization of TDP-43 in prostate cancer cells upon platinum drug treatments. Immunofluorescence staining of TDP-43 and Coilin showing the localization response to 24 hrs drug treatments in A) LNCaP, B) PC-3, and C) 22Rv1 cells. TDP-43 (green), Coilin (red), merge of TDP-43 and Coilin (Merge RG), brightfield, DAPI, and Merge of TDP-43, Coilin and DAPI is shown. Scale bar, 20 μm. Related to Figure 7.

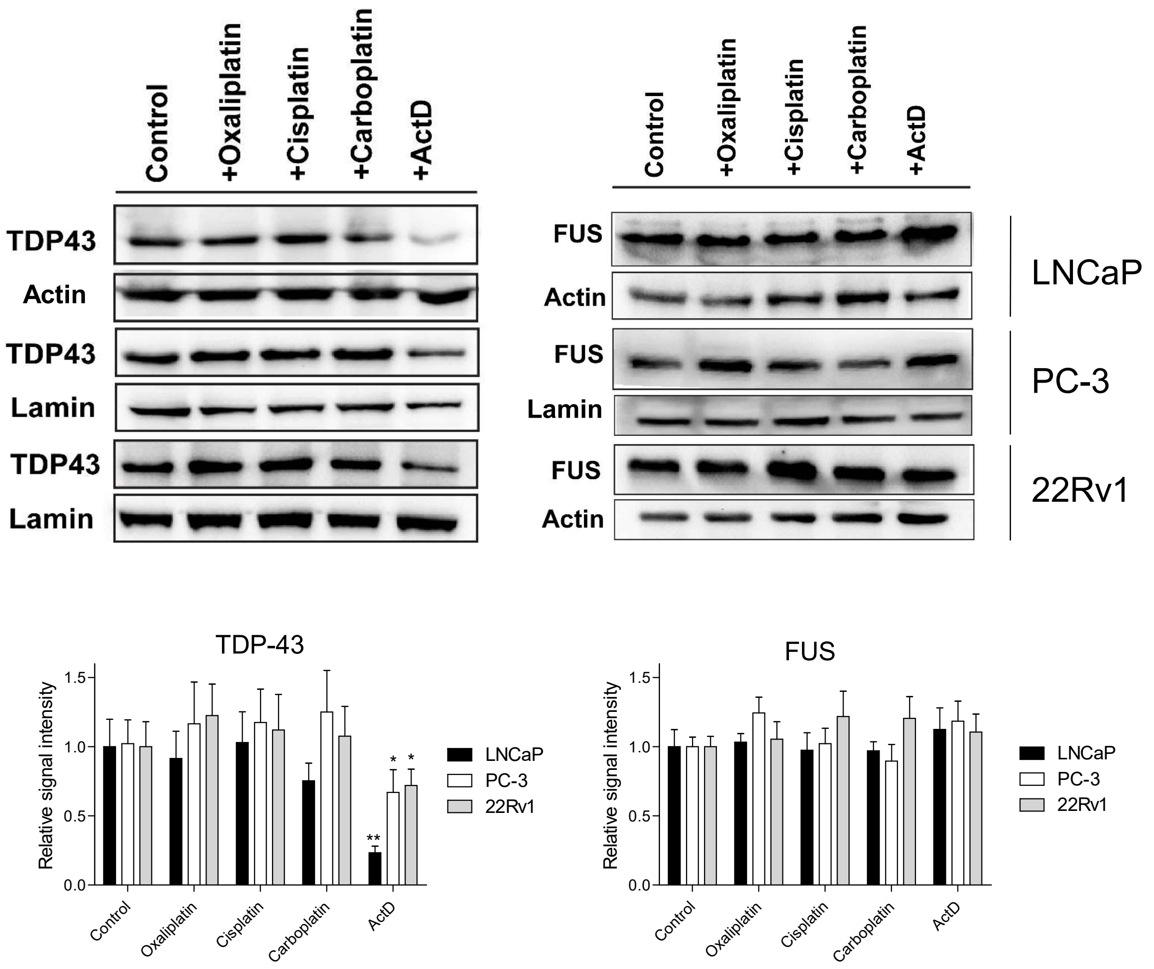

Additional file 1: Figure S7. Response of TDP-43 and FUS to platinum drugs in prostate cancer cells. Western blot analysis of TDP-43 (left p) and FUS (right panels) showing protein levels in response to 24 hrs drug treatments in the indicated cell lines. Lamin and actin are shown for loading controls. Upper panels, representative images of blots. Lower panels, relative signal intensity shown for three replicate experiments for each cell line. Mean values with S.D., *p-value <0.05, **p-value<0.01.

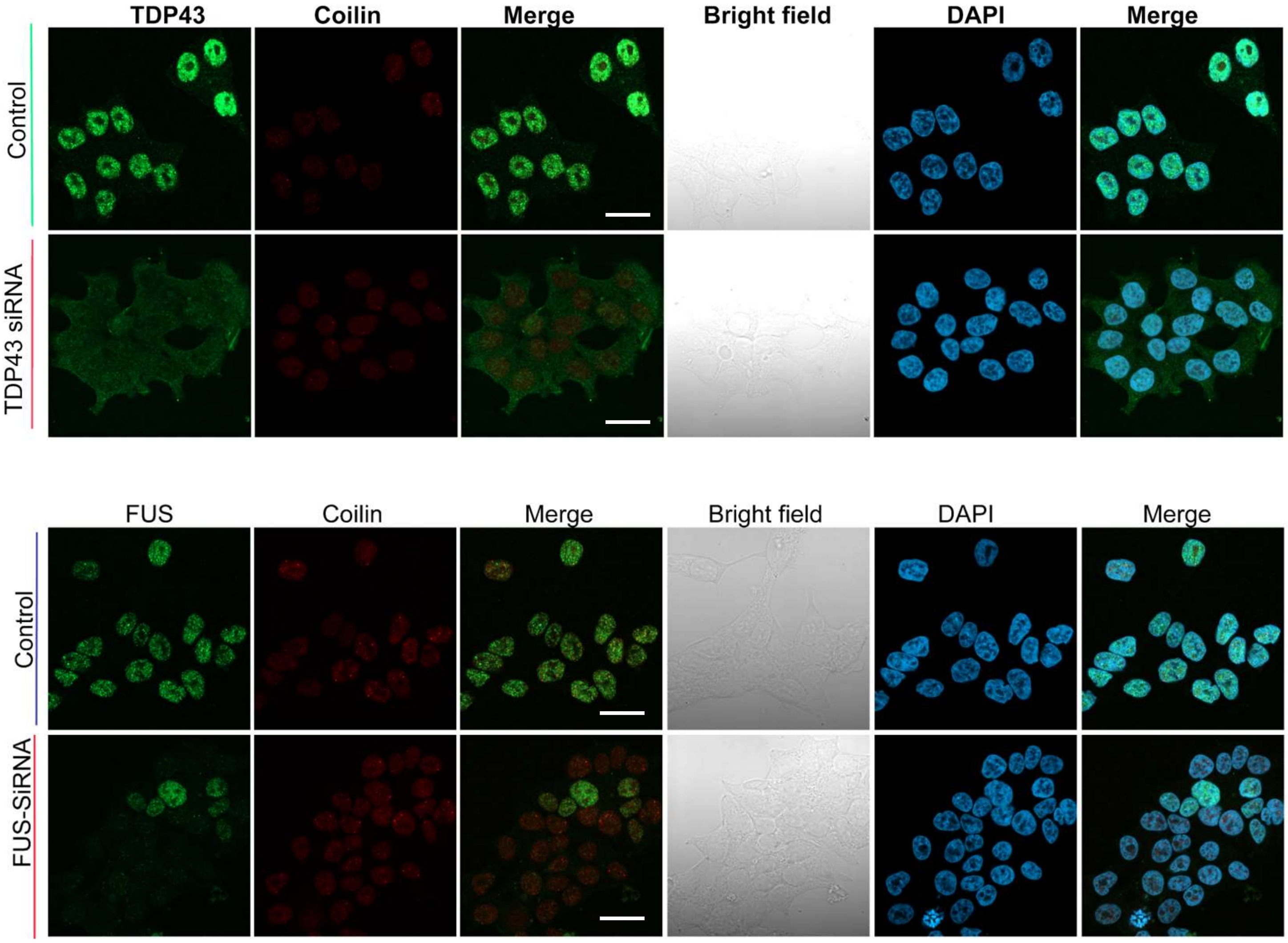

Additional file 1: Figure S8. Effect of TDP-43 and FUS in Coilin spots in 22Rv1 cells. Immunofluorescence analysis of localization of Coilin with TDP-43 and FUS upon siRNA downregulation ofA) TDP-43 and B) FUS in 22Rv1 cells. TDP-43 or FUS (green), Coilin (red), merge of TDP-43 or FUS and Coilin (Merge RG), brightfield, DAPI, and Merge of TDP-43 or FUS, Coilin and DAPI is shown. Scale bar, 20 μm. Related to Figure 8.

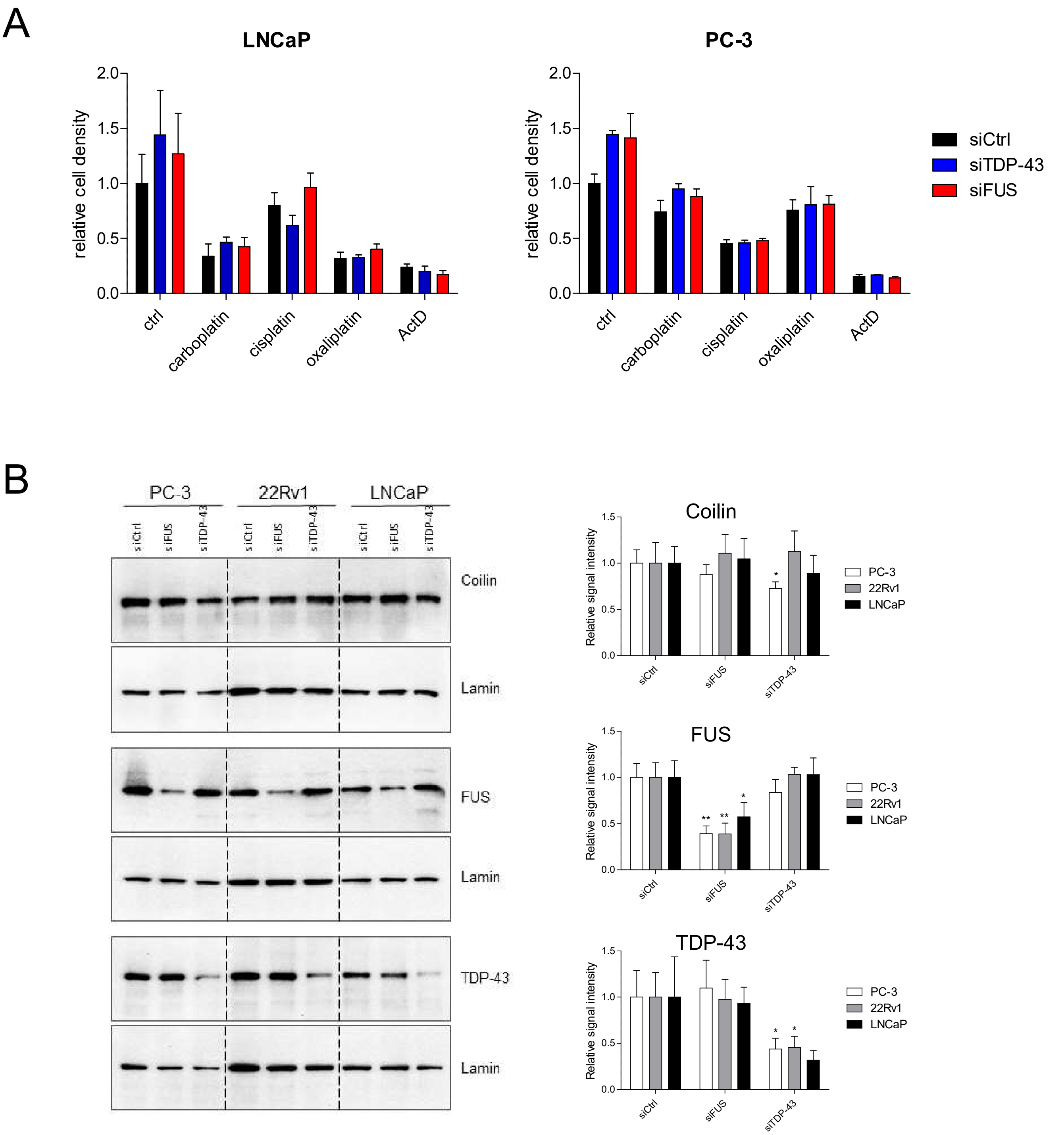

Additional file 1: Figure S9. Role of TDP-43 and FUS in platinum drug responses and regulation of Coilin in prostate cancer cells. A) Knock-down of TDP-43 and FUS with siRNA targeting in LNCaP and PC-3 cells followed by cell density measurements after 108 hrs of drug treatments. Error bars, SEM. B) Western blot analysis of Coilin, FUS and TDP-43 showing protein levels in response to 72 hrs siRNA treatments in the indicated cell lines. Lamin is used for loading control. Left panel, representative images of blots. Dashed lines indicate samples for each cell line. Right panel, relative signal intensity shown for three replicate experiments for each cell line. Mean values with S.D., *p-value <0.05, **p-value<0.01.
