# Supplementary material for: Platinum-based drugs induce phenotypic alterations in nucleoli and Cajal bodies in prostate cancer cells
Source: Cancer Cell Int. 2024 Jan 13;24:29. doi: 10.1186/s12935-023-03205-0 (PMC10790272; doi:10.1186/s12935-023-03205-0)
Supplement: Supplementary file 2 — Additional file 2: Table S1. Predominant nuclear signals of Cajal body proteins SMN1 and Coilin in cells treated with the indicated drugs for 24 h. CB, Cajal bodies. [file 12935_2023_3205_MOESM2_ESM.pdf]

Additional file2: Table S1 Predominanr nuclear signals of Cajal body proteins SMN1 and Coilin in cells treated with the indicated drugs for 24 hours. CB, Cajal bodies.

	**LNCaP**			**PC-3**			**22Rv1**		
	**SMN1**	**Coilin**	**Main site of colocalization**	**SMN1**	**Coilin**	**Main site of colocalization**	**SMN1**	**Coilin**	**Main site of colocalization**
**Control**	CB spot localization in ~50% of the cells	CB spot localization in ~50% of the cells	CB spots	CB spot localization in majority of the cells	CB spot localization in majority of the cells	CB spots	Low nuclear signal, no CB localization	CB spot localization in minority of the cells	No significant co-localization
**Oxaliplatin**	Decreased number and size of spots	Nucleolar capping	Nucleolar caps	Decreased number and size of spots	Increased number and decreased size of spots, nucleolar capping	Remaining CB spots	Low nuclear signal, no CB localization	Spots and nucleolar capping	No significant co-localization
**Cisplatin**	Spot localization lost	Nucleolar localization	No significant co-localization	Spot localization decreased	Nucleolar localization	Only occasional localization in nucleolar caps and remaining CB spots	Nucleolar localization in minority of the cells	Nucleolar localization	Occasional nucleoli
**Carboplatin**	Spot localization decreased	Decrease of signal	Remaining CB spots	Spot localization decreased	Decrease of signal	Remaining CB spots	Low nuclear signal, no CB localization	Decrease of signal	No significant co-localization
**ActD**	Spot localization lost	Nucleolar capping	Nucleolar caps	Spot localization lost	Nucleolar capping	Nucleolar caps	Nucleolar capping	Nucleolar capping	Nucleolar caps
